# Supplementary material for: Kinetically controlled hetero-fusion is a systems-level behaviour of polymer nanoparticle populations
Source: Nat Commun. 2025 Nov 28;16:11701. doi: 10.1038/s41467-025-66827-0 (PMC12753759; doi:10.1038/s41467-025-66827-0)
Supplement: Supplementary file 1 — Supplementary Information [file 41467_2025_66827_MOESM1_ESM.pdf]

Kinetically controlled hetero-fusion is a systems-level behaviour of polymer nanoparticle populations

Supplementary Information

Stephen D. P. Fielden,[1]* Sean M. Collins,[2,3] Matthew J. Derry,[4] Caterina Ducati,[5] Simon M. Fairclough,[5] Alisha J. Miller,[1] Rachel K. O'Reilly,[1] Paul D. Topham[4]

[1]School of Chemistry, University of Birmingham, Birmingham, UK
[2]School of Chemical and Process Engineering, University of Leeds, Leeds, UK
[3]Department of Materials, Imperial College London, London, UK
[4]Aston Institute for Membrane Excellence, Aston University, Birmingham, UK
[5]Department of Materials Science and Metallurgy, University of Cambridge, Cambridge, UK

*email: s.fielden@bham.ac.uk

Table of Contents

Supplementary Methods

1 General information and abbreviations........3

1.1 Materials........3

1.2 Abbreviations........3

1.3 Characterisation Techniques........4

2 Synthesis and characterisation of P(**NB-amine**)$_{11}$-*block*-P(**NB-MEG**)$_{200/300}$ (**A200/A300**) and P(**NB-PEG**)$_{11}$-*block*-P(**NB-MEG**)$_{100/150}$ (**P100/P150**)........6

3 Mixing **A200** and **P100** dispersions to produce **[A200•P100]**........13

4 Determining the extent of fusion........18

4.1 Calculation of fusion extents........18

4.2 Correlating fusion extent with the Carothers equation........23

5 SAXS analysis of hetero-fusion........25

5.1 SAXS Modelling........25

5.2 Static analyses........29

5.3 Static and time-resolved data........34

5.4 Time-resolved analyses........37

6 Calculation of particles per unit volume........39

7 Increasing MEG DP: Characterisation data for hetero-fused samples **[A200•P150]**, **[A300•P100]** and **[A300•P150]**........40

8 Synthesis of N**B-FluoroMEG**........42

9 Synthesis, characterisation and hetero-fusion of P(**NB-amine**)$_{11}$-*block*-P(**NB-FluoroMEG**)$_{200/300}$ (**AF200**)........44

10 Cryo-STEM-EELS analysis of **AF200** and **[AF200•P100]** particles........47

10.1 Experiment description........47

10.2 Data analysis........47

11 Additional control experiments........53

11.1 Storage stability study........53

11.2 Attempted hetero-fusion of **A200** and **P100** at low temperature........53

11.3 Increasing the THF content during hetero-fusion........54

11.4 Combining non-fusogenic particle populations **A100** and **P50**........55

11.5 Preliminary study of hetero-fusion at neutral pH........55

12 Supplementary References........57

Supplementary Methods

1 General information and abbreviations

1.1 Materials

Unless stated otherwise, reagents were obtained from commercial sources and used without purification. Tetrahydrofuran (THF) (HPLC grade) was purchased from VWR Chemicals and was purified via passage through a column of neutral alumina prior to use. Formvar-carbon coated (300 mesh) and lacey-carbon coated (400 mesh) copper grids were purchased from EM Resolutions.**G3** (modified Grubb's 3rd generation catalyst),[1] **NB-amine**,[2] **NB-MEG**,[2] and **NB-PEG**[3] were prepared as previously described.

1.2 Abbreviations

ADF	Annular Dark Field
CI	Chemical Ionisation
cryo	cryogenic
d	doublet
DLS	Dynamic Light Scattering
DMF	Dimethylformamide
DP	Degree of Polymerisation
EEL	Electron Energy Loss
EELS	Electron Energy Loss Spectroscopy
eq.	equivalents
HPLC	High Performance Liquid Chromatography
HRMS	High Resolution Mass Spectrometry
ICA	Independent Component Analysis
min	minute
N_{agg}	aggregation number
NMR	Nuclear Magnetic Resonance
q	quartet
P	Poly
PB2	Phosphate Buffer adjusted to pH2.
PMMA	poly(methyl methacrylate)
ROI	Region Of Interest
ROMPISA	Ring-Opening Metathesis Polymerisation-Induced Self-Assembly
rpm	rotations per minute
rt	room temperature
s	singlet
SAXS	Small Angle X-ray Scattering
SEC	Size Exclusion Chromatography
STEM	Scanning Transmission Electron Microscopy
t	triplet
TEM	Transmission Electron Microscopy
THF	Tetrahydrofuran
wt%	percentage by weight

1.3 Characterisation Techniques

NMR Spectroscopy ^{1}H, ^{19}F and ^{13}C NMR spectra were recorded on a Bruker DPX-300 or a Bruker DPX-400 spectrometer in $CDCl_3$, or CD_3CN. Chemical shifts of protons are reported as δ in parts per million (ppm) and are relative to solvent residual peaks ($CDCl_3$ δ = 7.26 ppm, CD_3CN δ = 1.94 ppm) All ^{1}H resonances are reported to the nearest 0.01 ppm. The multiplicity of ^{1}H signals are indicated as: s = singlet; d = doublet; t = triplet; q = quartet; m = multiplet; or combinations of thereof. Coupling constants (J) are quoted in Hz and reported to the nearest 0.1 Hz. Where appropriate, averages of the signals from peaks displaying multiplicity were used to calculate the value of the coupling constant. ^{13}C NMR spectra were recorded on the same spectrometer with the central resonance of the solvent peak as the internal reference ($CDCl_3$ δ = 77.16 ppm). All ^{13}C resonances are reported to the nearest 0.01 ppm.

High-Resolution Mass Spectrometry HRMS spectra were recorded by the Mass Spectrometry Facility Service at the University of Birmingham on a Thermo Scientific Orbitrap Exploris spectrometer.

Size Exclusion Chromatography Size exclusion chromatography (SEC) analysis was performed on a system composed of an Agilent 1260 Infinity II LC system equipped with an Agilent guard column (PLGel 5 µM, 50 × 7.5 mm) and two Agilent Mixed-C columns (PLGel 5 µM, 300 × 7.5 mm). The mobile phase used was THF (HPLC grade) containing 2% v/v NEt_3 at 40 °C with a flow rate of 1.0 mL min^{-1} (PMMA standards used for calibration). Number average molecular weight (M_n), weight average molecular weight (M_w) and dispersity ($Đ = M_w/M_n$) was determined using either Wyatt ASTRA v7.1.3 or Agilent GPC/SEC software.

Dynamic Light Scattering Hydrodynamic diameters (D_h) and size distributions (polydispersity, PD) of nano-objects were determined by dynamic light scattering (DLS) using a Malvern Zetasizer Nano ZS with a 4 mW He-Ne 633 nm laser module operating at 25 °C. Samples were diluted with PB2 to give a final polymer concentration of 0.01 wt%. Measurements were carried out at an angle of 173° (back scattering), and results were analysed using Malvern DTS v7.03 software. All determinations were repeated at least three times with at least 10 measurements recorded for each run. D_h values were calculated using the Stokes-Einstein equation where particles are assumed to be spherical, while for anisotropic particles DLS was used to indicate a change in average particle size and obtain dispersity information.

Transmission Electron Microscopy Dry-state stained transmission electron microscopy (TEM) imaging was performed on a JEOL JEM-1400 microscope operating at an acceleration voltage of 80 kV. All dry-state samples were diluted with PB2 to an appropriate analysis concentration and then deposited onto formvar-coated grids. After roughly 1 min, excess sample was blotted from the grid and the grid was then stained with an aqueous 1 wt% uranyl acetate (UA) or, where indicated, AGR1000 UA-Zero stain solution for 1 min prior to blotting, drying and microscopic analysis. Cryogenic transmission electron microscopy (cryo-TEM) imaging was performed on a JEOL JEM-2100Plus microscope operating at an acceleration voltage of 200 kV. Samples for cryo-TEM analysis were prepared, after dilution with PB2, by depositing 5 µL of sample onto a lacey carbon grid followed by blotting for approximately five seconds. The grid was then plunged into a pool of liquid ethane, cooled using liquid nitrogen, to vitrify the sample. Transfer into a pre-cooled cryo-TEM holder was performed under liquid nitrogen temperatures prior to microscopic analysis. Images were analysed using ImageJ. Cryo-EELS-STEM experimental details are included in section 10.

Small-angle X-ray scattering SAXS patterns were recorded at a synchrotron source (Diamond Light Source, station I22, Didcot, UK; Experiment ID SM33098) using monochromatic X-ray radiation (X-ray wavelength λ = 1.00 Å, with scattering vector q ranging from 0.0017 to 0.17 $Å^{-1}$, where $q = 4\pi \sin \theta/\lambda$ and θ is one-half of the scattering angle) and a 2D Pilatus 2M pixel detector (Dectris, Switzerland). All static SAXS measurements were performed on 1.0 wt% copolymer dispersions in 2.0 mm glass capillaries. Time-resolved experiments were performed using a BioLogic SFM-400 stopped-flow mixing system equipped with an umbilical connector, mixing unit and observation cell containing a 1.0 mm glass capillary, without delay lines. Particle dispersions of **A200** and **P100** (1.0 wt%) were loaded into two separate syringes before 200 µL of each solution was flowed into the observation cell at 1.0 mL s^{-1}. Scattering data were reduced and normalized, with glassy carbon being used for the absolute intensity calibration, utilising standard routines available at the beamline.[4]

2 Synthesis and characterisation of P(NB-amine)$_{11}$-*block*-P(NB-MEG)$_{200/300}$ (A200/A300) and P(NB-PEG)$_{11}$-*block*-P(NB-MEG)$_{100/150}$ (P100/P150)

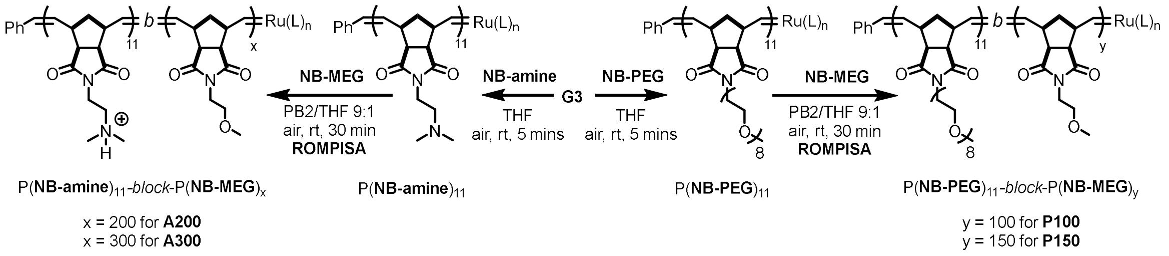

Supplementary Figure 1. Synthesis of A200, A300, P100 and P150. Synthetic scheme of ROMPISA.

A solution of **NB-amine** (11.6 mg, 50 µmol, 11 eq.) or **NB-PEG** (26.3 mg, 50 µmol, 11 eq.) in 900 µL filtered THF was rapidly added to a solution of **G3** (3.3 mg, 4.5 µmol, 1.0 eq.) in 100 µL filtered THF, contained within a 2 mL glass vial equipped with a stirrer bar. The resulting solutions were stirred rapidly for five minutes.

An aliquot (50 µL for **A200**, 33 µL for **A300**, 100 µL for **P100** or 67 µL for **P150**) of macroinitiator solution in THF was dispensed into a 2 mL glass vial containing a stirrer bar. Filtered THF was added to give 100 µL total volume in each vial. A solution of **NB-MEG** (10 mg, 45 µmol) in 0.9 mL of acidic phosphate buffer (pH = 2, PB2, final solids concentration = 1 wt%) was added rapidly to each vial. The resulting solution was thoroughly mixed by drawing up the entire volume into the pipette tip and ejecting the liquid back into the vial three times. The ROMPISA polymerisations were stirred at 300 rpm for 30 minutes to give **A200, A300, P100** and **P150** nanoparticle populations. These were analysed by ^{1}H NMR, SEC, DLS, TEM and SAXS. Aliquots analysed by SEC were quenched by addition of several drops of ethyl vinyl ether.

Characterisation Summary

Sample	$M_{n,theo}$ /kDa	$M_{n,SEC}$[a] /kDa	$Đ_{SEC}$	$D_{h,DLS}$ /nm	PD_{DLS}	L_{TEM} /nm
P(**NB-amine**)$_{11}$	2.6	3.1	1.15	n/a	n/a	n/a
P(**NB-PEG**)$_{11}$	5.8	6.5	1.13	n/a	n/a	n/a
**A200**	47	38	1.11	36	0.12	29 ± 5
**A300**	69	50	1.12	50	0.10	44 ± 14
**P100**	28	31	1.09	33	0.12	28 ± 5
**P150**	39	39	1.12	44	0.09	43 ± 13

[a]THF eluent, PMMA standards

Supplementary Table 1 Characterisation of unmixed polymers and nanoparticles.

^{1}H NMR

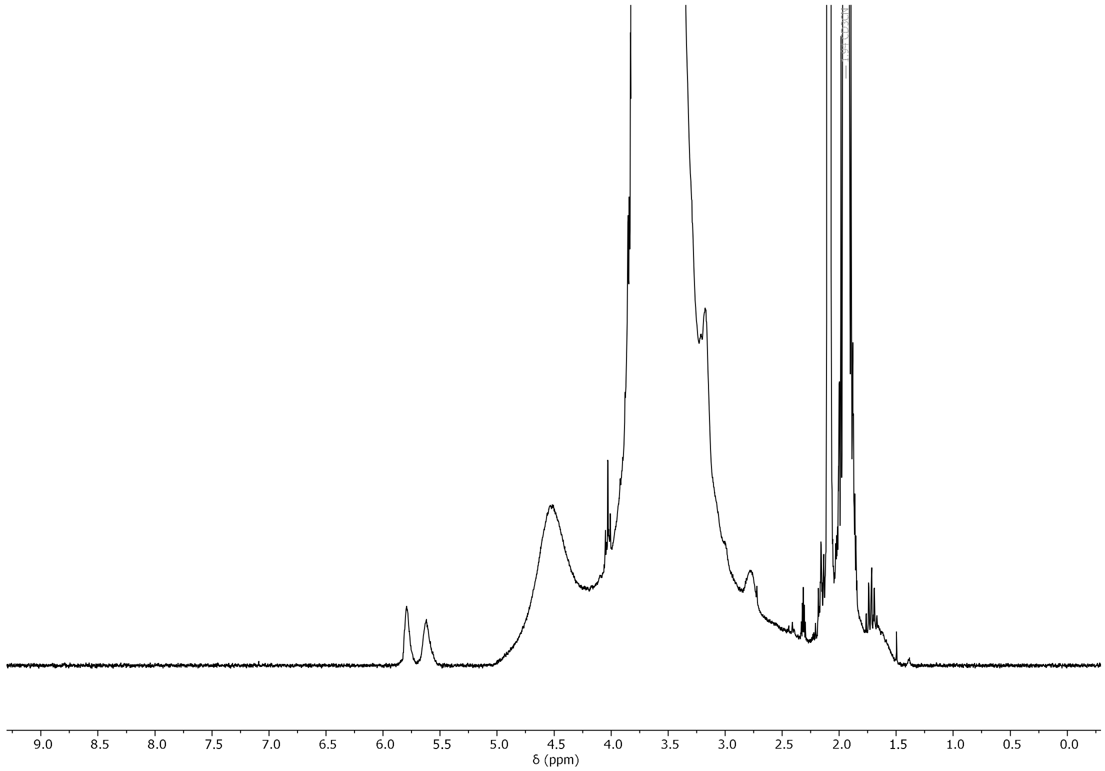

Supplementary Figure 2. NMR spectrum of A200. ^{1}H NMR (300 MHz, CD_3CN, 300 K) of **A200** + THF/PB2

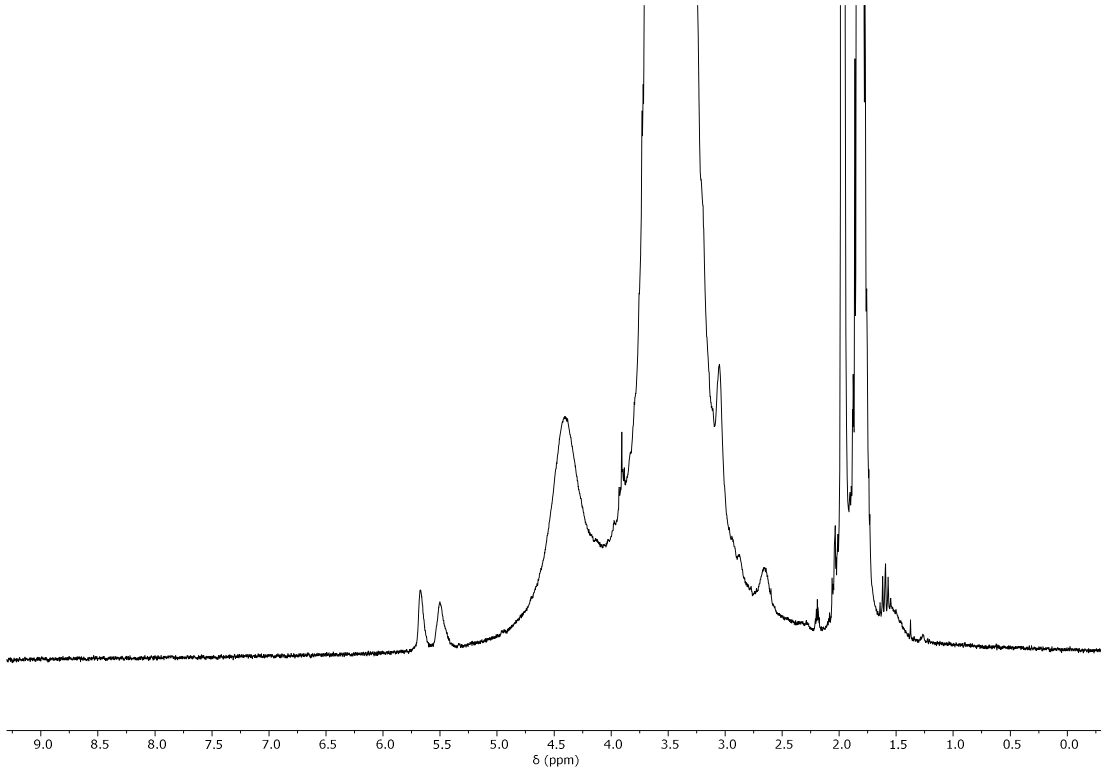

Supplementary Figure 3. NMR spectrum of A300. ^{1}H NMR (300 MHz, CD_3CN, 300 K) of **A300** + THF/PB2

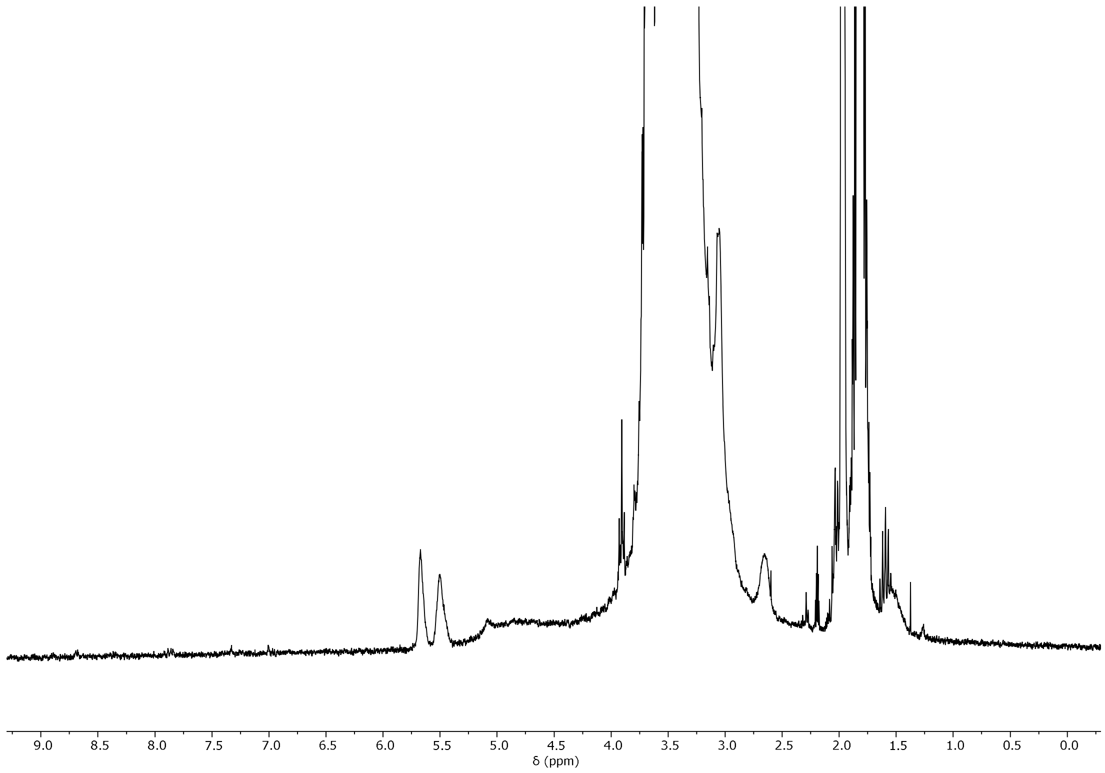

Supplementary Figure 4. NMR spectrum of P100. ^{1}H NMR (300 MHz, CD_3CN, 300 K) of **P100** + THF/PB2

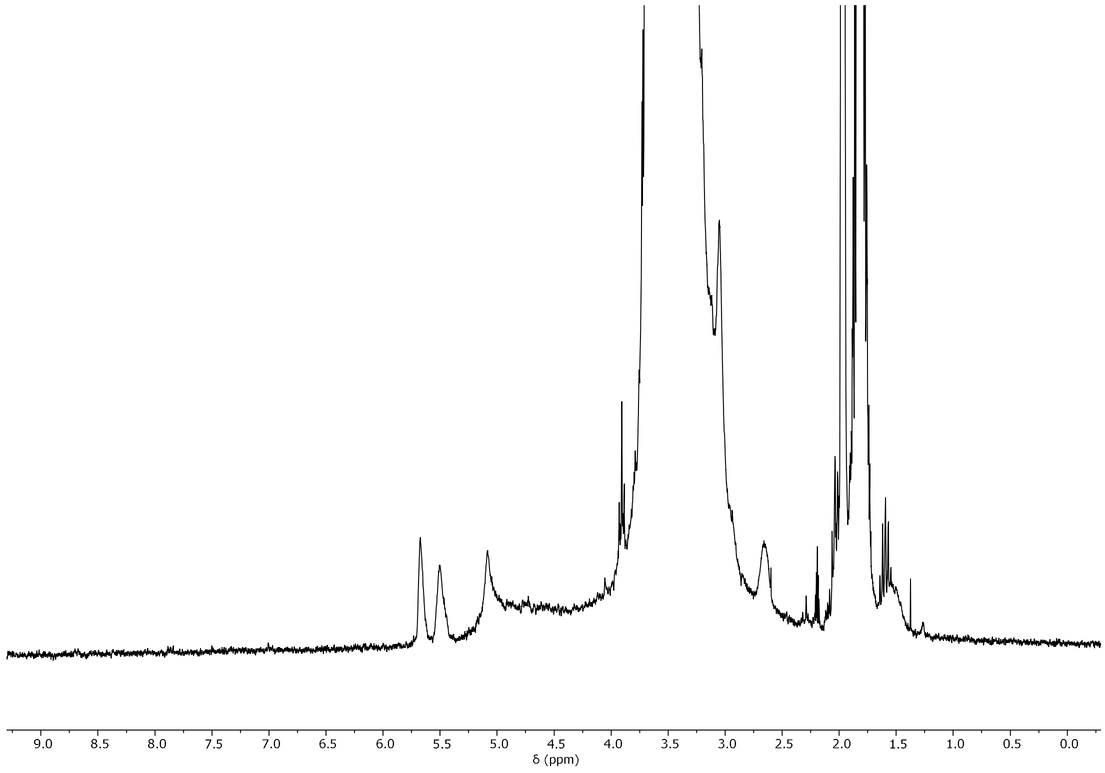

Supplementary Figure 5. NMR spectrum of P150. ^{1}H NMR (300 MHz, CD_3CN, 300 K) of **P150** + THF/PB2

SEC

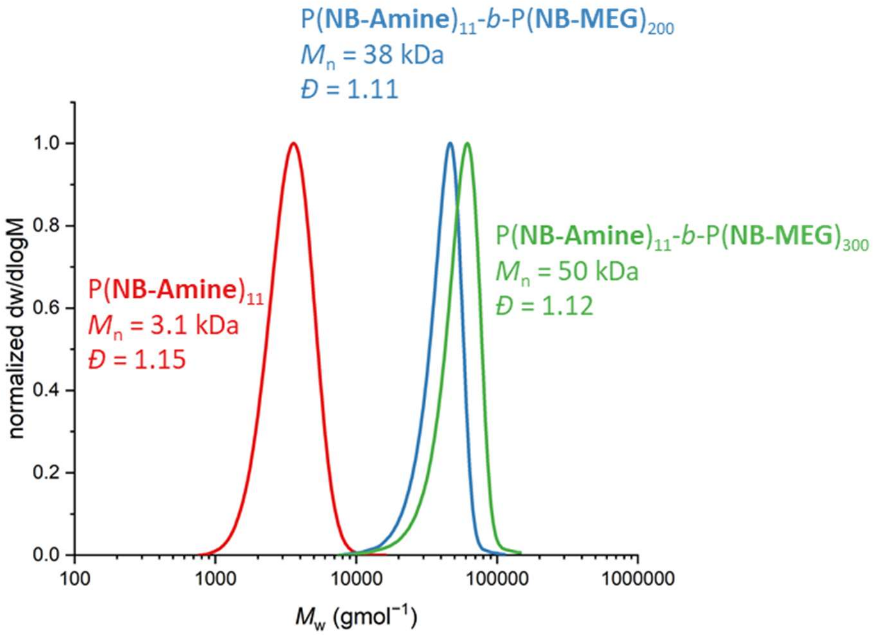

Supplementary Figure 6. SEC data for NB-amine polymers. Normalized SEC trace (THF eluent, PMMA standards) of P(**NB-amine**)$_{11}$, **A200** and **A300**.

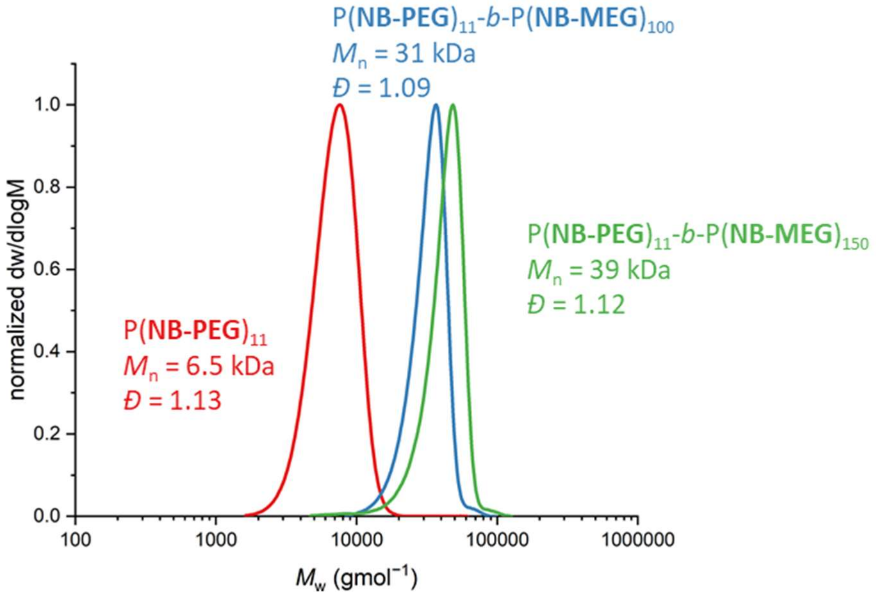

Supplementary Figure 7. SEC data for NB-PEG polymers. Normalized SEC trace (THF eluent, PMMA standards) of P(**NB-PEG**)$_{11}$, **P100** and **P150**.

DLS

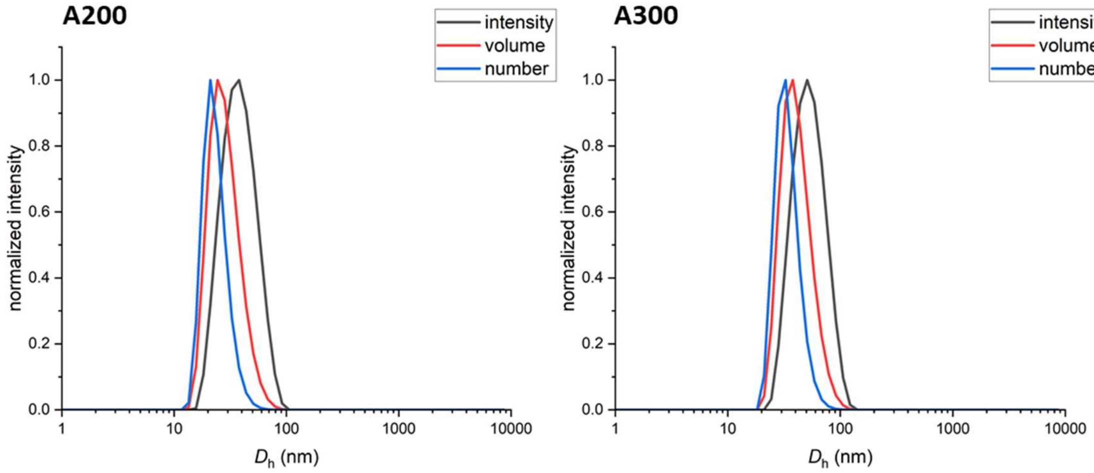

Supplementary Figure 8. DLS data for NB-amine particles. DLS traces of **A200** and **A300** particles

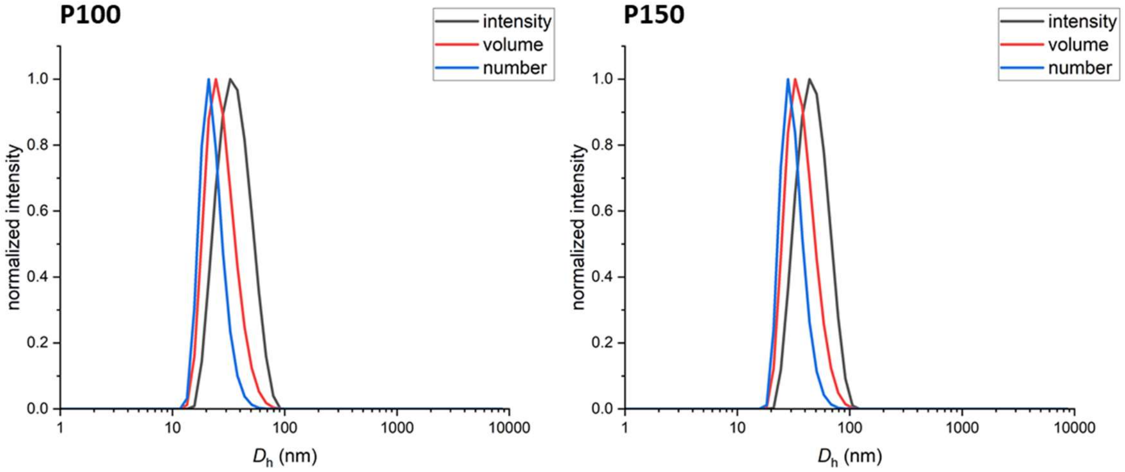

Supplementary Figure 9. DLS data for NB-PEG particles. DLS traces of **P100** and **P150** particles

Dry-state TEM

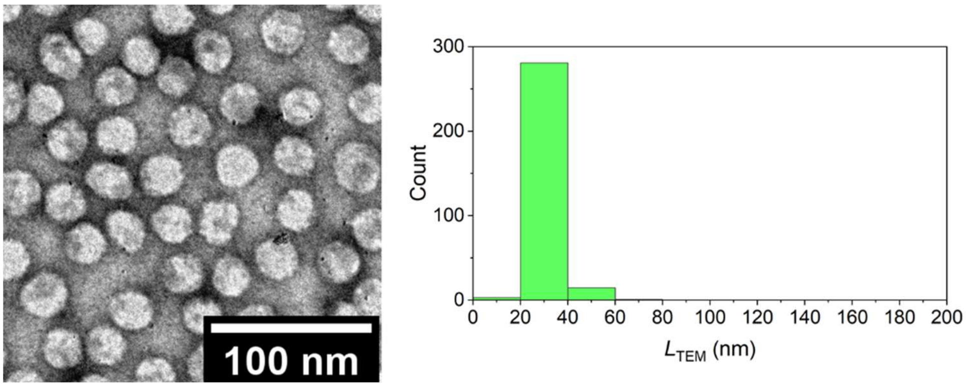

Supplementary Figure 10. TEM data of A200 particles. Dry-state TEM image and histogram (300 particles analysed) of **A200** particles

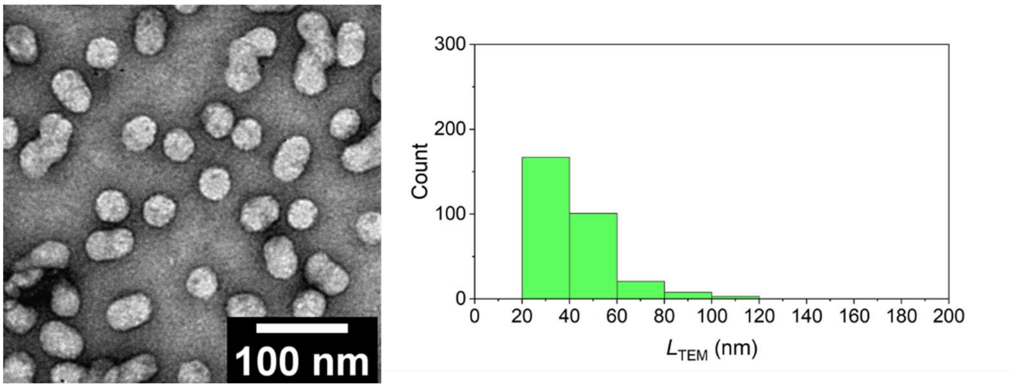

Supplementary Figure 11. TEM data of A300 particles. Dry-state TEM image and histogram (300 particles analysed) of **A300** particles

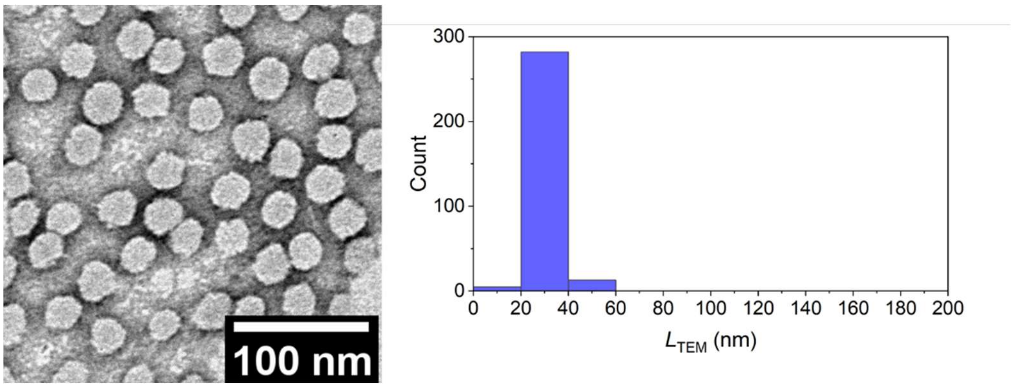

Supplementary Figure 12. TEM data of P100 particles. Dry-state TEM image and histogram (300 particles analysed) of **P100** particles

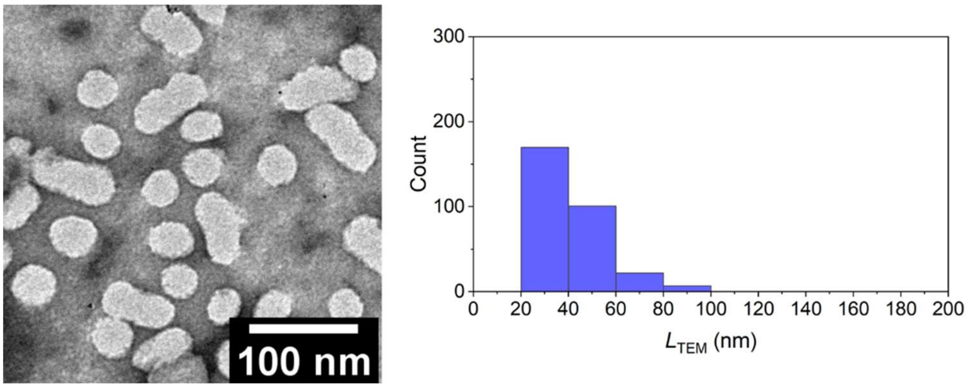

Supplementary Figure 13. TEM data of P150 particles. Dry-state TEM image and histogram (300 particles analysed) of **P150** particles

Cryo-TEM

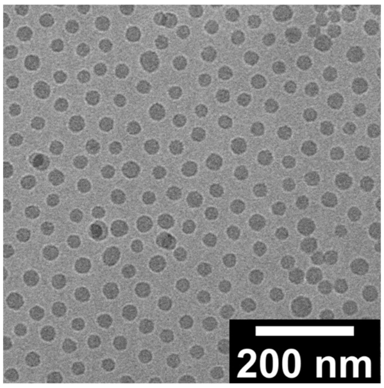

Supplementary Figure 14. Cryo-TEM data of A200 particles. Cryo-TEM image of **A200** particles

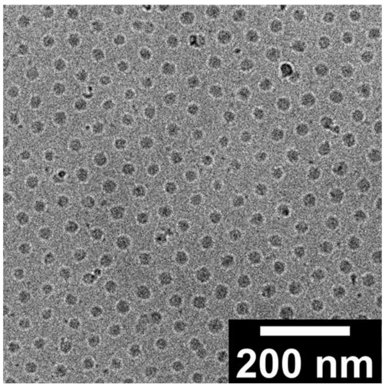

Supplementary Figure 15. Cryo-TEM data of P100 particles. Cryo-TEM image of **P100** particles

3 Mixing A200 and P100 dispersions to produce [A200•P100]

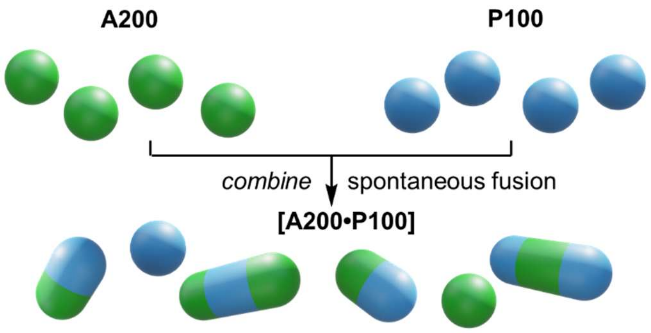

Supplementary Figure 16. Combining A200 and P100 dispersions to produce [A200•P100] Cartoon depicting mixing process to produce fused particles.

An aliquot of **A200** was added to a 2 mL glass vial by pipette. A second aliquot of **P100** was added into the vial to produce a final volume of 200 µL. The resulting mixture was thoroughly mixed by drawing up the entire volume into the pipette tip and ejecting the liquid back into the vial three times. The mixture was left for 30 minutes without stirring before analysis.

Characterisation Summary

Volume ratio **A200**:**P100** dispersions	$D_{h,DLS}$ /nm	PD_{DLS}	L_{TEM} /nm
**P100 only**	33	0.12	28 ± 5
1:9	39	0.12	33 ± 8
2:8	43	0.14	-
3:7	49	0.15	38 ± 13
4:6	54	0.12	-
**5:5 (1:1)**	**55**	**0.17**	**45 ± 23**
6:4	61	0.14	-
7:3	63	0.15	50 ± 26
8:2	60	0.15	-
9:1	51	0.16	36 ± 14
**A200 only**	36	0.12	29 ± 5

Supplementary Table 2 Characterisation of hetero-fused samples derived from **A200** and **P100**

DLS

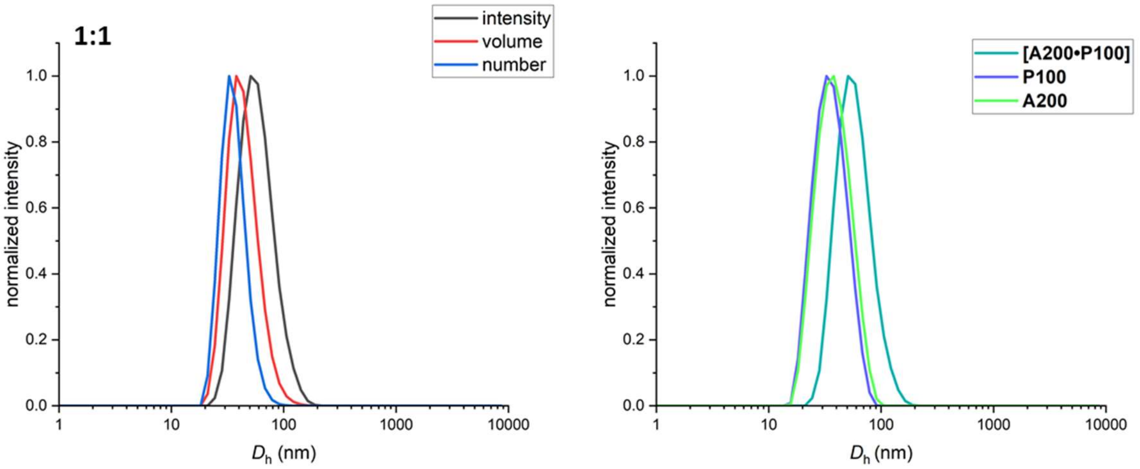

Supplementary Figure 17. DLS data for [A200•P100] particles at equal volume ratio. DLS traces of **[A200•P100]** particles at 1:1 **A200**:**P100** volume ratio and intensity-weighted DLS traces of **A200**, **P100** and **[A200•P100]** particles

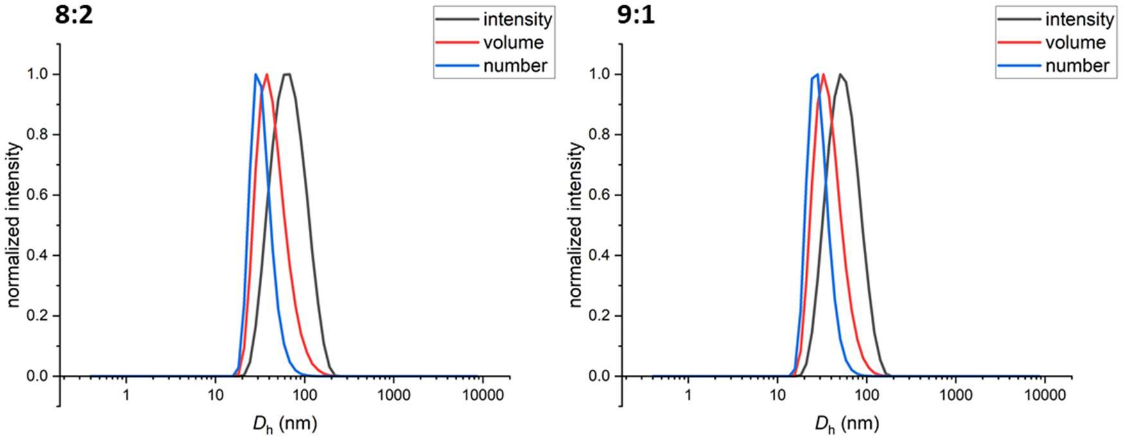

Supplementary Figure 18. DLS data for [A200•P100] particles with 80% or 90% A200 content. DLS traces of **[A200•P100]** particles at 8:2 and 9:1 **A200**:**P100** volume ratio

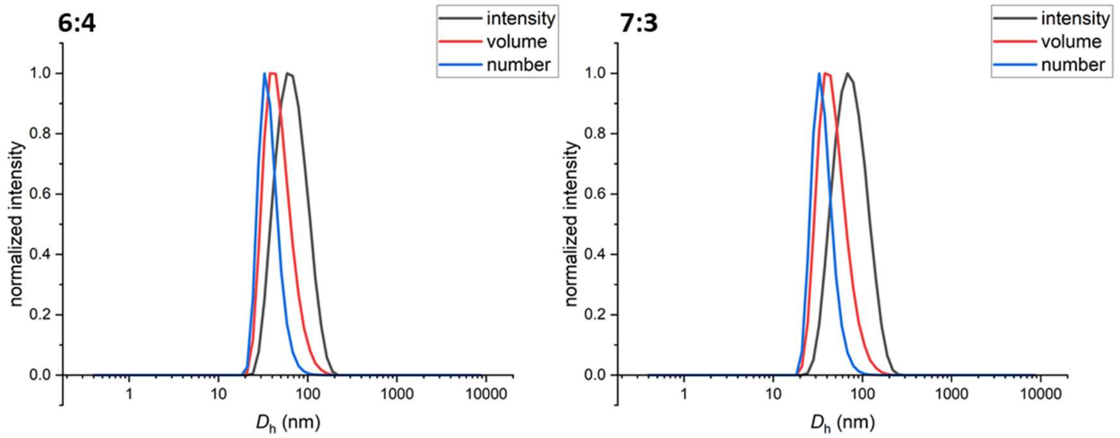

Supplementary Figure 19. DLS data for [A200•P100] particles with 60% or 70% A200 content. DLS traces of **[A200•P100]** particles at 6:4 and 7:3 **A200**:**P100** volume ratio

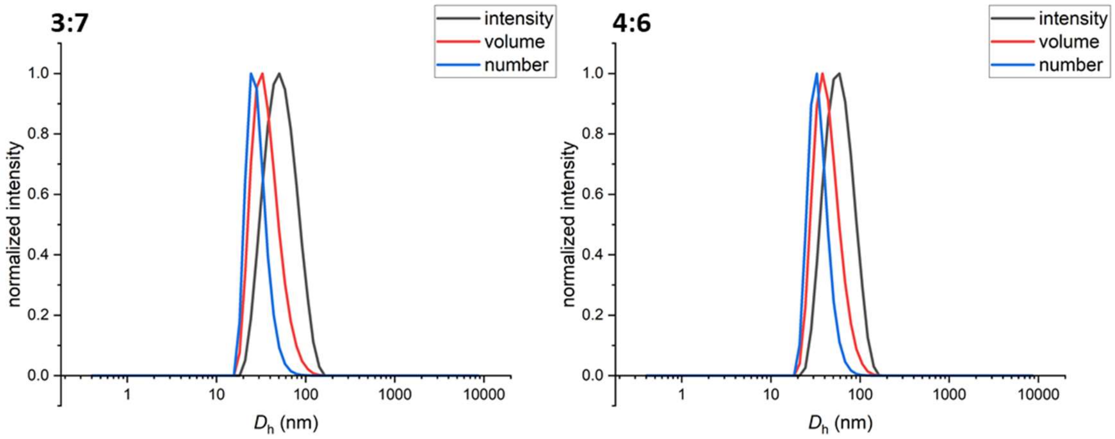

Supplementary Figure 20. DLS data for [A200•P100] particles with 30% or 40% A200 content. DLS traces of **[A200•P100]** particles at 3:7 and 4:6 **A200**:**P100** volume ratio

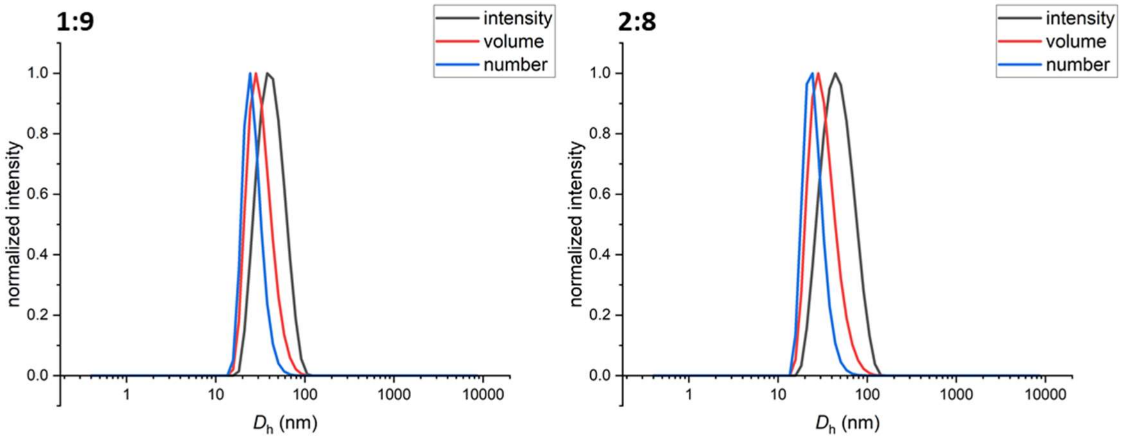

Supplementary Figure 21. DLS data for [A200•P100] particles with 10% or 20% A200 content. DLS traces of **[A200•P100]** particles at 1:9 and 2:8 **A200**:**P100** volume ratio

Dry-state TEM

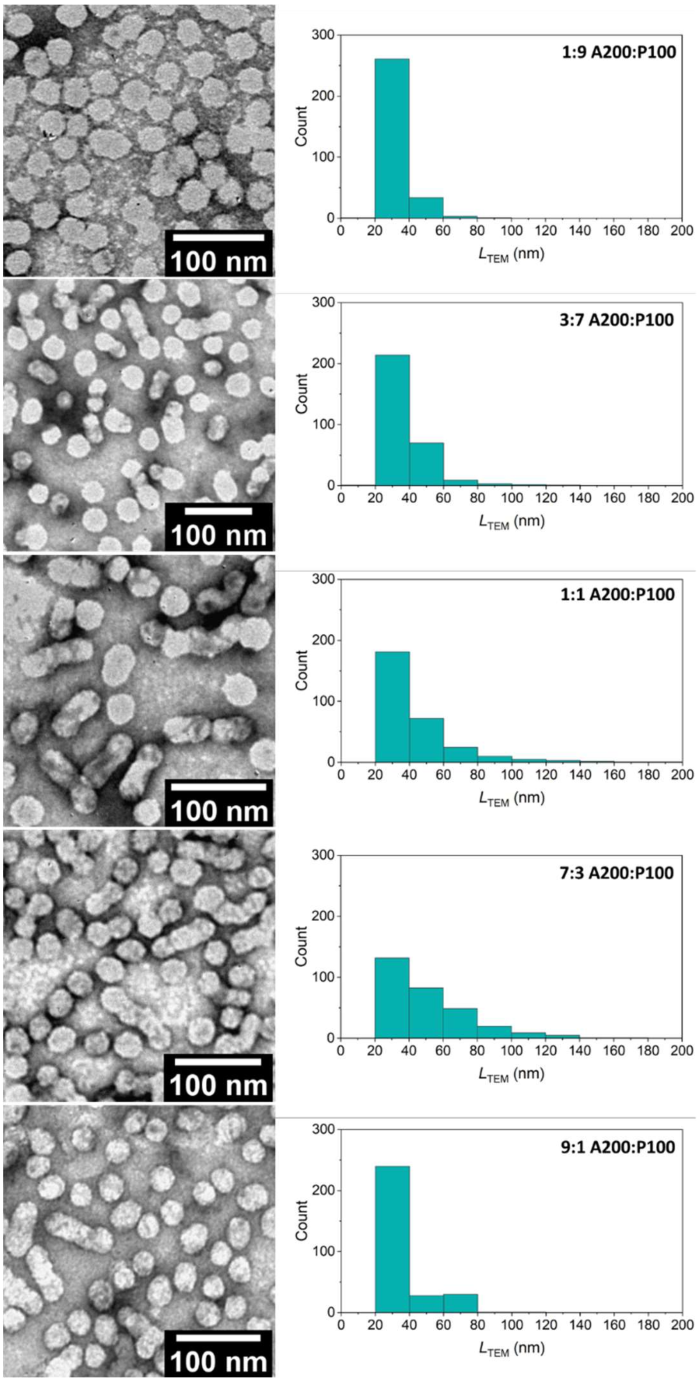

Supplementary Figure 22. TEM data for [A200•P100] particles. Dry-state TEM images and histograms (300 particles per sample analysed) of **[A200•P100]** samples containing 10%, 30%, 50%, 70% and 90% volume fraction of **A200** dispersions.

Cryo-TEM

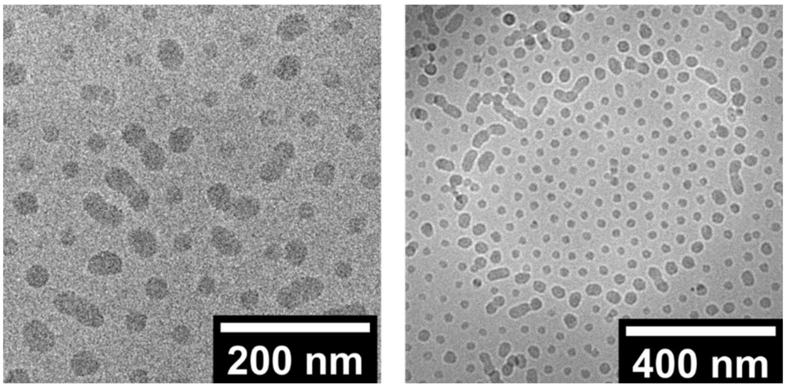

Supplementary Figure 23. Cryo-TEM data for [A200•P100] particles. Cryo-TEM images of **[A200•P100]** particles at 1:1 **A200**:**P100** volume ratio at close and far zoom. Note the apparent self-sorting of particles, presumably due to variations in amorphous ice thickness.

4 Determining the extent of fusion

4.1 Calculation of fusion extents

Calculation of fusion extent for [A200•P100] 1:1 volume ratio

- A representative distribution of **[A200•P100]** particles was obtained by measuring the length of 300 particles by dry-state TEM. This sample includes unfused spheres and fused particles of varying length. Fused particles are formed from an integer number of spheres.

- The average unfused particle length is 28.3 nm.

- The number of spherical particles required to form each fused particle can be estimated by binning the particles into ranges of multiples of the average unfused length. For example, a fused particle formed from two particles has length 57 ± 14 nm. The binned results are as follows:

Constituent spherical particles	Length threshold (nm)	Number of particles counted	Total constituent spherical particles
1 (unfused)	14-42	196	196
2	42-71	76	152
3	71-99	16	48
4	99-127	6	24
5	127-156	4	20
6	156-184	2	12
>6	>184	0	0
	total	300	452

Supplementary Table 3 Composition of **[A200•P100]** 1:1 volume ratio

- The total number of spherical particles required to form the 300 counted particles is determined by multiplying the number of spheres required to form each particle length by the number of particles counted.

- Total number of spheres = 452
  Number of fused spheres = 256
  Number of unfused spheres = 196

- Degree of fusion = number of fused spheres/total number of spheres = 256/452 = 0.57 (57%)

Calculation of fusion extent for [A200•P100] 7:3 volume ratio

Constituent spherical particles	Length threshold (nm)	Number of particles counted	Total constituent spherical particles
1 (unfused)	14-43	140	140
2	43-71	102	204
3	71-100	42	126
4	100-128	12	48
5	128-157	3	15
6	157-185	1	6
>6	>185	0	0
	total	300	539

Supplementary Table 4 Composition of **[A200•P100]** 7:3 volume ratio

- Total number of spheres = 539
  Number of fused spheres = 399
  Number of unfused spheres = 140

- Degree of fusion = number of fused spheres/total number of spheres = 399/539 = 0.74 (74%)

Calculation of fusion extent for [A200•P100] 3:7 volume ratio

Constituent spherical particles	Length threshold (nm)	Number of particles counted	Total constituent spherical particles
1 (unfused)	14-43	229	229
2	43-71	59	118
3	71-100	9	27
4	100-128	3	12
>4	>128	0	0
	total	300	386

Supplementary Table 5 Composition of **[A200•P100]** 3:7 volume ratio

- Total number of spheres = 386
  Number of fused spheres = 157
  Number of unfused spheres = 229

- Degree of fusion = number of fused spheres/total number of spheres = 157/386 = 0.41 (41%)

Calculation of fusion extent for [A200•P100] 9:1 volume ratio

Constituent spherical particles	Length threshold (nm)	Number of particles counted	Total constituent spherical particles
1 (unfused)	14-43	245	245
2	43-71	43	86
3	71-100	11	33
4	100-128	1	4
>5	>128	0	0
	total	300	368

Supplementary Table 6 Composition of **[A200•P100]** 9:1 volume ratio

- Total number of spheres = 368
  Number of fused spheres = 123
  Number of unfused spheres = 245

- Degree of fusion = number of fused spheres/total number of spheres =123/368 = 0.19 (33%)

Calculation of fusion extent for [A200•P100] 1:9 volume ratio

Constituent spherical particles	Length threshold (nm)	Number of particles counted	Total constituent spherical particles
1 (unfused)	14-43	270	270
2	43-71	27	54
3	71-100	3	9
>3	100-128	0	0
	total	300	333

Supplementary Table 7 Composition of **[A200•P100]** 1:9 volume ratio

- Total number of spheres = 333
  Number of fused spheres = 63
  Number of unfused spheres = 270

- Degree of fusion = number of fused spheres/total number of spheres =63/333 = 0.19 (19%)

Calculation of fusion extent for A300 unmixed particles

Average width = 32 ± 3 nm; average length = 44 ± 14 nm

In spherical particles width equals length. Therefore, average width = length of average unfused particle.

Constituent spherical particles	Length threshold (nm)	Number of particles counted	Total constituent spherical particles
1 (unfused)	16-48	208	208
2	48-79	81	162
3	79-111	9	27
4	111-143	2	8
>4	>143	0	0
	total	300	405

Supplementary Table 8 Composition of **A300** unmixed particles

- Total number of spheres = 405
  Number of fused spheres = 197
  Number of unfused spheres = 208

- Degree of fusion = number of fused spheres/total number of spheres = 197/405 = 0.49 (49%)

Calculation of fusion extent for P150 unmixed particles

Average width = 33 ± 4 nm; average length = 43 ± 13 nm

In spherical particles width equals length. Therefore, average width = length of average unfused particle.

Constituent spherical particles	Length threshold (nm)	Number of particles counted	Total constituent spherical particles
1 (unfused)	17-50	234	234
2	50-83	62	124
3	83-116	4	12
>3	>116	0	0
	total	300	370

Supplementary Table 9 Composition of **P150** unmixed particles

- Total number of spheres = 370
  Number of fused spheres = 136
  Number of unfused spheres = 234

- Degree of fusion = number of fused spheres/total number of spheres = 136/370 = 0.37 (37%)

Calculation of fusion extent for [A300•P150] particles

- The average particle length before hetero-fusion is 43.3 nm.

Constituent starting particles	Length threshold (nm)	Number of particles counted	Total constituent starting particles
1 (no-hetero fusion)	22-65	83	83
2	65-108	80	160
3	108-152	48	144
4	152-195	39	156
5	195-238	28	140
6	238-282	11	66
7	282-325	8	56
8	325-368	1	8
9	368-412	0	0
10	412-455	1	10
11	455-498	1	11
>11	>498	0	0
	total	300	834

Supplementary Table 10 Composition of **[A300•P150]** 1:1 volume ratio

- Total number of starting particles = 834
  Number of cross fused starting particles = 751
  Number of non-cross fused starting particles = 83

- Degree of fusion = number of cross-fused particles/total number of starting particles = 751/834 = 0.90 (90%)

4.2 Correlating fusion extent with the Carothers equation

- The Carothers equation correlates number average degree of polymerisation, X, with monomer consumption, p: $X = \frac{1}{1-p}$

- When considering particle fusion, spherical particles are regarded as 'monomers' and cylinders as 'polymers'.

- X is calculated by dividing the number average particle length after fusion with the number average unfused particle length (i.e., dividing polymer length by monomer length).

- p is calculated by: $p = \frac{N_o - N}{N_o}$, where N_o = number of particles at the start of the reaction and N = number of particles at the end of the reaction (Supplementary Table 11).

- Here, N = 300 (the number of particles measured in each TEM sample) and N_o is calculated by determining the number of spherical particles needed to form the resultant 300 particles. For example, when **A200** and **P100** dispersions are mixed in a 1:1 volume ratio, N_o = 449.

Volume ratio of **A200**:**P100** dispersions	p	$X_{theoretical}$	$X_{observed}$	$X_{theoretical} - X_{observed}$
7:3	0.443	1.80	1.78	0.02
1:1	0.336	1.51	1.60	−0.09
3:7	0.222	1.29	1.36	−0.07
9:1	0.185	1.23	1.28	−0.05
1:9	0.099	1.11	1.16	−0.05
1:1 **A300**:**P150**	0.640	2.78	2.78	0

Supplementary Table 11 Comparing degrees of polymerisation with monomer consumption using the unmodified Carothers equation.

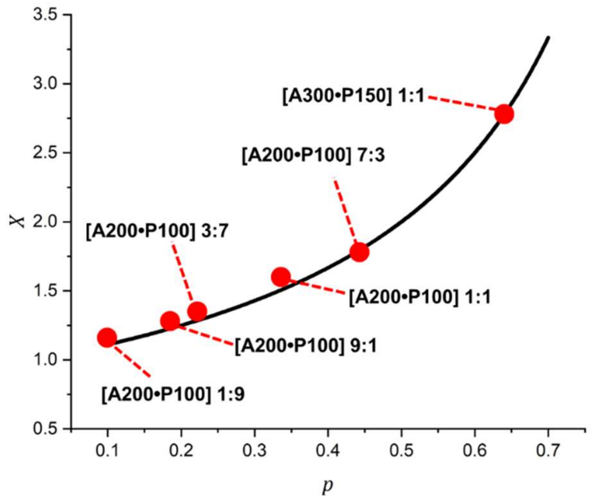

Supplementary Figure 24. Plotting fusion degree to the Carothers Equation. Plot of average degree of polymerisation versus monomer consumption. Red spots are values determined for different hetero-fusion samples (volume ratios indicated) and the black line is the theoretical fit given by the Carothers equation.

- Note that the Carothers equation is valid for a polymerisation of two monomers when they are present in equal quantities (i.e. there is no stoichiometric excess). When there is an imbalance, $r = \frac{N_{limiting}}{N_{excess}}$, where $N_{limiting}$ is given by the amount of limiting reagent and N_{excess} is given by the amount of excess reagent, which means $r \leq 1$, the modified Carothers equation becomes:[5] $X = \frac{1+r}{1+r-2rp}$

- The imbalance, r, can be calculated for **A200**:**P100** from converting volume ratios to number ratios of particles (Section 6, values shown in Supplementary Table 12). This leads to different values of $X_{theoretica}$.

Volume ratio of **A200**:**P100** dispersions	r	p	$X_{theoretical}$	$X_{observed}$	$X_{theoretical} - X_{observed}$
7:3	0.64	0.443	1.53	1.78	−0.25
1:1	0.67	0.336	1.37	1.60	−0.23
3:7	0.22	0.222	1.11	1.36	−0.25
9:1	0.17	0.185	1.06	1.28	−0.22
1:9	0.074	0.099	1.01	1.16	−0.15

Supplementary Table 12 Comparing degrees of polymerisation with monomer consumption using the unmodified Carothers equation.

- As can be seen from Supplementary Table 12, the match of $X_{theoretica}$ and $X_{observed}$ is significantly worse than that for the unmodified equation (Supplementary Table 11). This suggests that not all particles are capable of undergoing fusion (i.e., are fusogenic). In other words, r is, in reality, closer to 1 than as calculated from total particle numbers. The reason for this remains unclear.

5 SAXS analysis of hetero-fusion

5.1 SAXS Modelling

Programming tools within the Irena SAS macros for Igor Pro were used to model experimental SAXS data.[6] Models used to fit SAXS data in this work were previously developed by Pedersen *et al.*[7]

In general, the intensity of X-rays scattered by a dispersion of nano-objects [as represented by the scattering cross-section per unit sample volume, $\frac{d\Sigma}{d\Omega}(q)$] can be expressed as:

$$\frac{d\Sigma}{d\Omega}(q) = NS(q)\int_0^\infty \dots \int_0^\infty F(q, r_{1,} \dots, r_k)^2 \Psi(r_{1,} \dots, r_k) dr_{1,} \dots, dr_k \qquad \text{(S1)}$$

where $F(q, r_1, \dots, r_k)$ is the form factor, $r_{1,} \dots, r_k$ is a set of *k* parameters describing the nano-object structural morphology, $\Psi(r_{1,} \dots, r_k)$ is the distribution function, *S*(*q*) is the structure factor and *N* is the number density of nano-objects per unit volume expressed as:

$$N = \frac{\varphi}{\int_0^\infty \dots \int_0^\infty V(r_1, \dots, r_k)\Psi(r_1, \dots, r_k) dr_1, \dots, dr_k} \qquad \text{(S2)}$$

where $V(r_1, \dots, r_k)$ is the nano-object volume and φ is the volume fraction of the nano-objects within the dispersion. It is assumed that *S*(*q*) = 1 at the sufficiently low copolymer concentrations used in this study (≤1.0% w/w).

For this study, experimental SAXS data required fitting to either the spherical micelle model, cylindrical micelle, or a combination of the two. Additionally, optimum fits often required the use a power law relationship (applied with radius of gyration cut off of 500 Å, herein described as a unified fit) and/or a low-intensity background to adequately fit low-*q* and high-*q* data, respectively. Thus, the intensity of scattering at a given *q* vector, $I(q)$, is expressed as:

$$I(q) = \frac{d\Sigma}{d\Omega}(q)_{\text{sphere}} + \frac{d\Sigma}{d\Omega}(q)_{\text{cylinder}} + Bq^{-P} + background \qquad \text{(S3)}$$

where $\frac{d\Sigma}{d\Omega}(q)_{\text{sphere}}$ is the form factor for spherical micelles, $\frac{d\Sigma}{d\Omega}(q)_{\text{cylinder}}$ is the form factor for cylindrical micelles, and terms B, P and $background$ are constants.

Spherical micelle model

The spherical micelle form factor for Equation S1 which contributes to $\frac{d\Sigma}{d\Omega}(q)_{\text{sphere}}$, is given by:

$$F_{\text{S}_{\text{mic}}}(q) = N_{\text{s}}{}^2\beta_{\text{s}}{}^2A_{\text{s}}{}^2(q,R_{\text{s}}) + N_{\text{s}}\beta_{\text{c}}{}^2F_{\text{c}}(q,R_{\text{g}}) + N_{\text{s}}(N_{\text{s}}-1)\beta_{\text{c}}{}^2A_{\text{c}}{}^2(q) + 2N_{\text{s}}{}^2\beta_{\text{s}}\beta_{\text{c}}A_{\text{s}}(q,R_{\text{s}})A_{\text{c}}(q) \quad \text{(S4)}$$

where R_{s} is the volume-average sphere core radius and R_{g} is the radius of gyration of the coronal steric stabilizer block (poly(**NB-amine**) or poly(**NB-PEG**). The X-ray scattering length contrasts for the core and corona blocks are given by $\beta_{\text{s}} = V_{\text{s}}(\xi_{\text{s}} - \xi_{\text{sol}})$ and $\beta_{\text{c}} = V_{\text{c}}(\xi_{\text{c}} - \xi_{\text{sol}})$ respectively. Here, ξ_{s}, ξ_{c} and ξ_{sol} are the X-ray scattering length densities of the core block (ξ_{s}= 10.77 x 10^{10} cm^{-2}), corona block (ξ_{c}= 10.31 x 10^{10} cm^{-2}) and water (ξ_{sol}= 9.42 x 10^{10} cm^{-2}), respectively. V_{s} and V_{c} are the volumes of the core block and the corona block, respectively. Values for V_{s} and V_{c} were calculated using $V = \frac{M_{\text{n}_{\text{pol}}}}{N_{\text{A}}\rho}$ taking the solid-state homopolymer densities of P(**NB-MEG**) determined by helium pycnometry ($\rho_{\text{P}(\textbf{NB-MEG})}$ = 1.19 g cm^{-3}) and P(**NB-PEG**) and P(**NB-amine**), which was taken to equal that of PEG ($\rho_{\text{P}(\textbf{NB-PEG})}$ = 1.13 g cm^{-3}),[8] where $M_{\text{n}_{\text{pol}}}$ is the number-average molecular weight of each polymer block determined by ^{1}H NMR spectroscopy. The sphere form factor amplitude is used for the amplitude of the core self-term:

$$A_{\text{c}}(q,R_{\text{s}}) = \Phi(qR_{\text{s}})\exp\left(-\frac{q^2\sigma^2}{2}\right) \quad \text{(S5)}$$

where $\Phi(qR_{\text{s}}) = \frac{3[\sin(qR_{\text{s}}) - qR_{\text{s}}\cos(qR_{\text{s}})]}{(qR_{\text{s}})^3}$. A sigmoidal interface between the two blocks was assumed for the spherical micelle form factor (Equation S4). This is described by the exponent term with a width σ accounting for a decaying scattering length density at the micellar interface. This σ value was fixed at 2.2 during fitting.

The form factor amplitude of the spherical micelle corona is:

$$A_{\text{c}}(q) = \frac{\int_{R_{\text{s}}}^{R_{\text{s}}+2s}\mu_c(r)\frac{\sin(qr)}{qr}r^2dr}{\int_{R_{\text{s}}}^{R_{\text{s}}+2s}\mu_c(r)r^2dr}\exp\left(-\frac{q^2\sigma^2}{2}\right) \quad \text{(S6)}$$

The radial profile, $\mu_{\text{c}}(r)$, can be expressed by a linear combination of two cubic b splines, with two fitting parameters s and a corresponding to the width of the profile and the weight coefficient respectively. This information can be found elsewhere,[9,10] as can the approximate integrated form of Equation S5. The self-correlation term for the coronal block is given by the Debye function:

$$F_c(q, R_g) = \frac{2[\exp(-q^2 R_g{}^2) - 1 + q^2 R_g{}^2]}{q^4 R_g{}^4} \quad \text{(S7)}$$

where R_g is the radius of gyration of the poly(**NB-PEG**) or poly(**NB-amine**) coronal block. The aggregation number, N_s, of the spherical micelle is given by:

$$N_s = (1 - x_{sol}) \frac{\frac{4}{3}\pi R_s{}^3}{V_s} \quad \text{(S8)}$$

where x_{sol} is the volume fraction of solvent within the poly(**NB-MEG**) micelle cores, which was found to be zero in all cases. A polydispersity for one parameter (R_s) is assumed for the micelle model, which is described by a Gaussian distribution. Thus, the polydispersity function in Equation S1 can be represented as:

$$\Psi(r_1) = \frac{1}{\sqrt{2\pi\sigma_{R_s}{}^2}} \exp\left(-\frac{(r_1 - R_s)^2}{2\sigma_{R_s}{}^2}\right) \quad \text{(S9)}$$

where σ_{R_s} is the standard deviation for R_s. In accordance with Equation S2, the number density per unit volume for the micelle model is expressed as:

$$N = \frac{\varphi_s}{\int_0^\infty V(r_1)\Psi(r_1)dr_1} \quad \text{(S10)}$$

where φ_s is the total volume fraction of copolymer in the spherical micelles and $V(r_1)$ is the total volume of copolymer within a spherical micelle $[V(r_1) = (V_s + V_c)N_s(r_1)]$.

Cylindrical micelle model

The cylindrical micelle form factor for Equation S1 is given by:

$$F_{cyl}(q) = N_{cyl}{}^2\beta_s{}^2F_{sw}(q) + N_w\beta_c{}^2F_c(q, R_g) + N_{cyl}(N_{cyl} - 1)\beta_c{}^2S_{cc}(q) + 2N_{cyl}{}^2\beta_s\beta_cS_{sc}(q) \quad \text{(S11)}$$

where all the parameters are the same as those described in the spherical micelle model (Equation S4), unless stated otherwise.

The self-correlation term for the cylinder core cross-sectional volume-average radius R_{cyl} is:

$$F_{\mathrm{cyl}}(q) = F_{\mathrm{cyl}}(q, L_{\mathrm{cyl}}, b_{\mathrm{cyl}}) {A_{\mathrm{CS}_{\mathrm{cyl}}}}^2(q, R_{\mathrm{cyl}}) \tag{S12}$$

where

$$ {A_{\mathrm{CS}_{\mathrm{cyl}}}}^2(q, R_{\mathrm{cyl}}) = \left[2 \frac{J_1(qR_{\mathrm{cyl}})}{qR_{\mathrm{cyl}}} \right]^2 \tag{S13}$$

and J_1 is the first-order Bessel function of the first kind, and a form factor $F_{\mathrm{cyl}}(q, L_{\mathrm{cyl}}, b_{\mathrm{cyl}})$ for self-avoiding semi-flexible chains represents the cylindrical micelles, where b_{cyl} is the Kuhn length and L_{cyl} is the mean contour length. In all cases when applying the cylindrical micelle model, b_{cyl} was found to equal the value of L_{cyl}, indicating the presence of rigid cylinders. A complete expression for the chain form factor can be found elsewhere.[11]

The mean aggregation number of the cylindrical micelle, N_{cyl}, is given by:

$$N_{\mathrm{cyl}} = (1 - x_{\mathrm{sol}}) \frac{\pi {R_{\mathrm{cyl}}}^2 L_{\mathrm{cyl}}}{V_{\mathrm{s}}} \tag{S14}$$

Again, x_{sol} was found to be zero in all cases. The possible presence of semi-spherical caps at both ends of each worm is neglected in this form factor.

A polydispersity for one parameter (R_{cyl}) is assumed for the cylindrical micelle model, which is described by a Gaussian distribution. Thus, the polydispersity function in Equation S1 can be represented as:

$$\Psi(r_1) = \frac{1}{\sqrt{2\pi {\sigma_{R_{\mathrm{cyl}}}}^2}} \exp\left(- \frac{(r_1 - R_{\mathrm{cyl}})^2}{2{\sigma_{R_{\mathrm{cyl}}}}^2} \right) \tag{S15}$$

where $\sigma_{R_{\mathrm{cyl}}}$ is the standard deviation for R_{cyl}. In accordance with Equation S2, the number density per unit volume for the worm-like micelle model is expressed as:

$$N = \frac{\varphi_{\mathrm{cyl}}}{\int_0^\infty V(r_1)\Psi(r_1) dr_1} \tag{S16}$$

where φ_{cyl} is the total volume fraction of copolymer in the cylindrical micelles and $V(r_1)$ is the total volume of copolymer in a cylindrical micelle $\left[V(r_1) = (V_{\mathrm{s}} + V_{\mathrm{c}}) N_{\mathrm{cyl}}(r_1) \right]$.

5.2 Static analyses

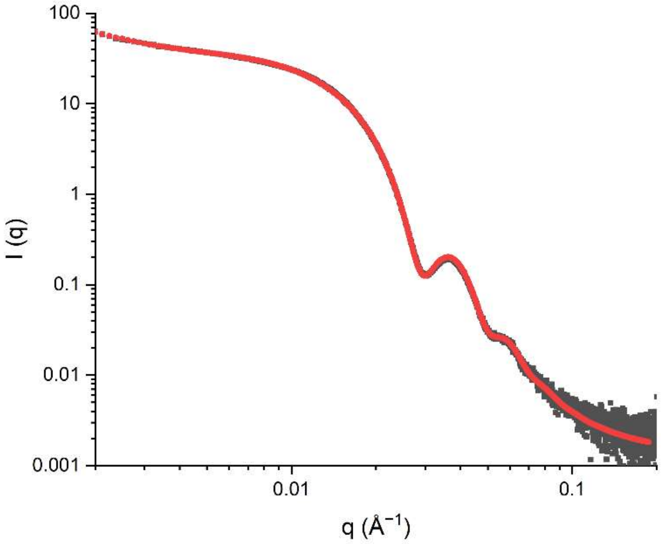

Supplementary Figure 25. SAXS plot for A200 particles. SAXS data for **A200** particles (black scatter) and fit to spherical micelle model (red line).

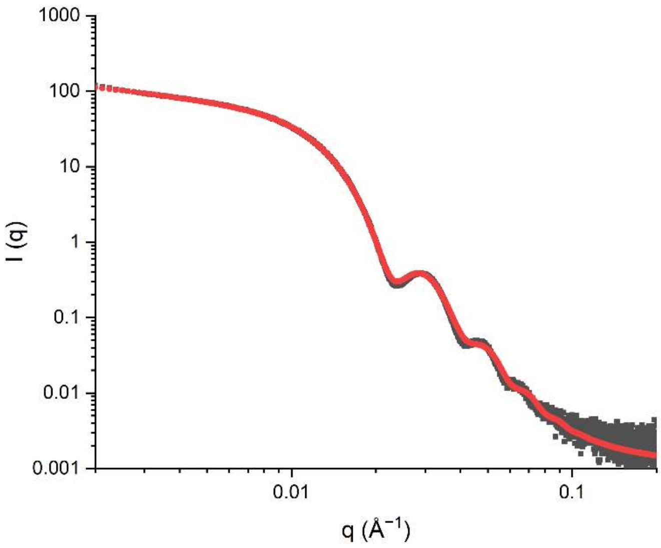

Supplementary Figure 26. SAXS plot for A300 particles. SAXS data for **A300** particles (black scatter) and fit to spherical micelle model (red line).

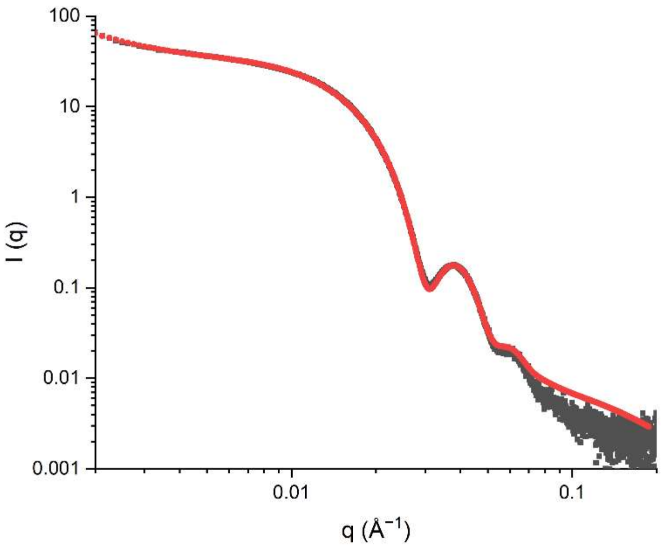

Supplementary Figure 27. SAXS plot for P100 particles. SAXS data for **P100** particles (black scatter) and fit to spherical micelle model (red line).

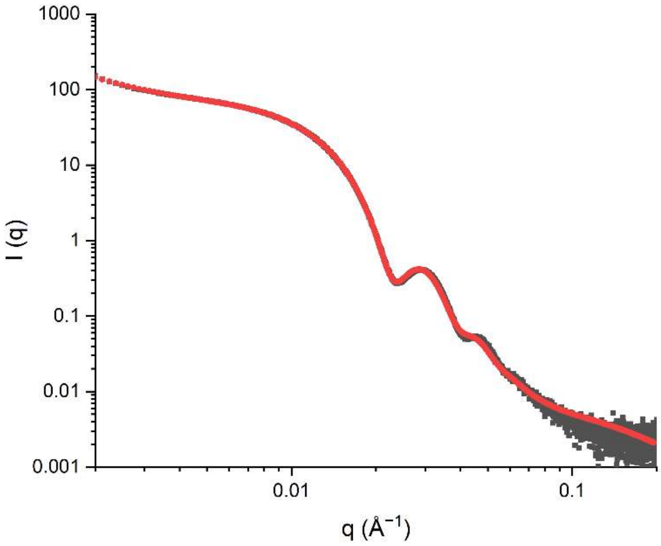

Supplementary Figure 28. SAXS plot for P150 particles. SAXS data for **P150** particles (black scatter) and fit to spherical micelle model (red line).

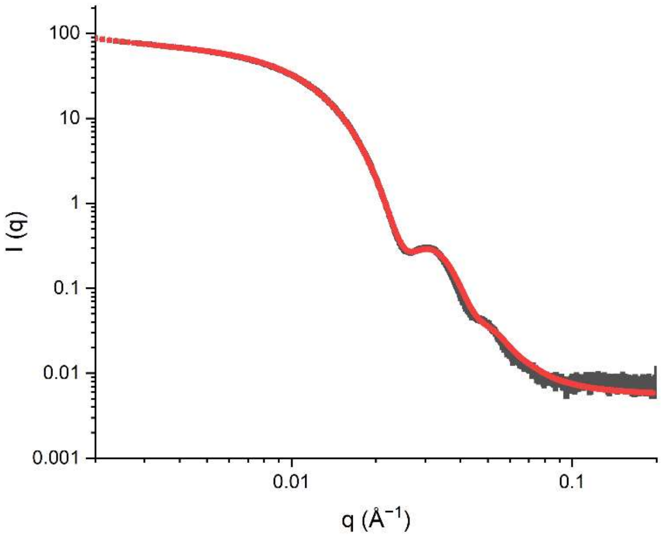

Supplementary Figure 29. SAXS plot for [A200•P100] particles fitted to spherical and cylindrical models. SAXS data for **[A200•P100]** particles formed from a 1:1 volume ratio of **A200** and **P100** particles (black scatter) and fit to a combination of the spherical micelle and cylindrical micelle models (red line).

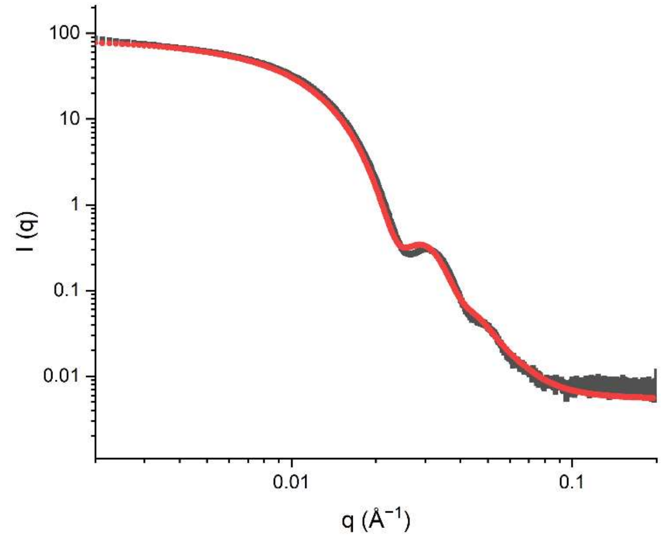

Supplementary Figure 30. SAXS plot for [A200•P100] particles fitted to spherical, dimer, trimer models SAXS data for **[A200•P100]** particles formed from a 1:1 volume ratio of **A200** and **P100** particles (black scatter) and fit to the sphere, dimer and trimer model (red line).[12] This model was judged to worse than the hard sphere and cylinder model so was not used further.

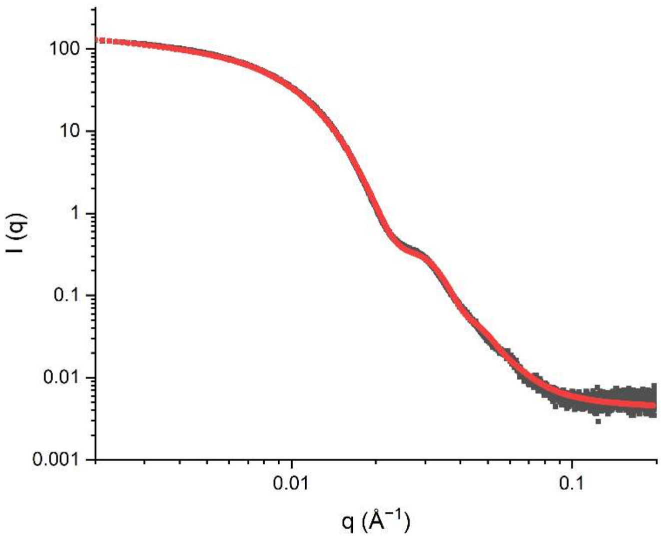

Supplementary Figure 31. SAXS plot for [A200•P150] particles. SAXS data for **[A200•P150]** particles (black scatter) and fit to a combination of the spherical micelle and cylindrical micelle models (red line).

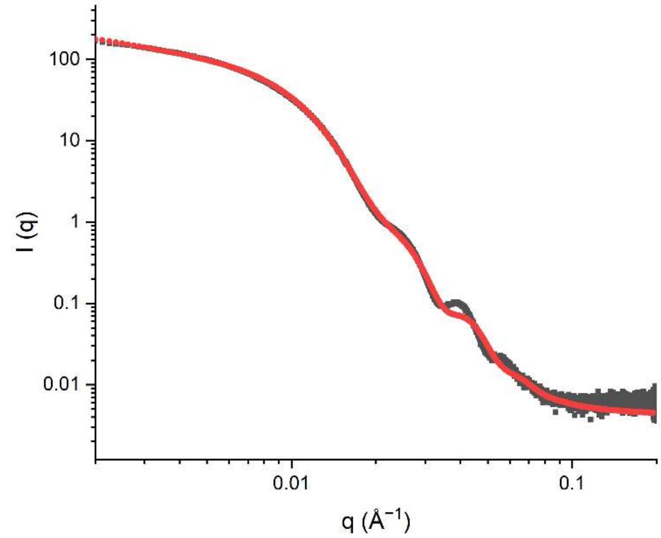

Supplementary Figure 32. SAXS plot for [A300•P100] particles. SAXS data for **[A300•P100]** particles (black scatter) and fit to a combination of the spherical micelle and cylindrical micelle models (red line).

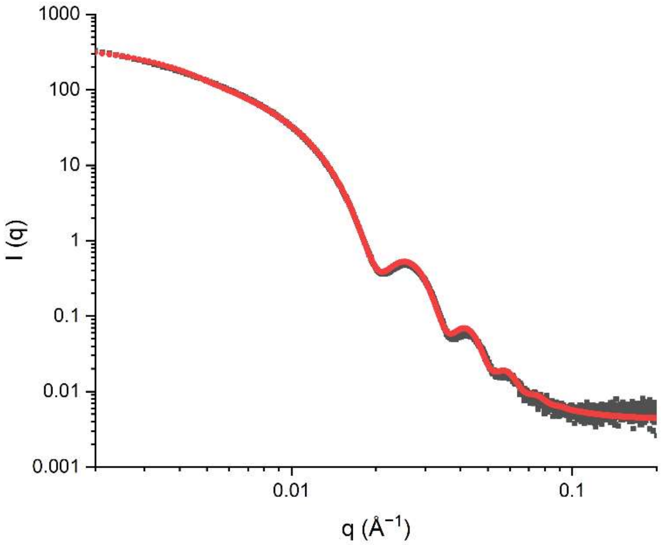

Supplementary Figure 33. SAXS plot for [A300•P150] particles. SAXS data for **[A300•P150]** particles (black scatter) and fit to a combination of the spherical micelle and cylindrical micelle models (red line).

5.3 Static and time-resolved data

Sample	Spherical micelle model			Cylindrical micelle model				Power law		$background$
	φ_{s}	R_{s} (Å)	$\sigma_{R_{\mathrm{s}}}$ (Å)	φ_{cyl}	R_{cyl} (Å)	$\sigma_{R_{\mathrm{cyl}}}$ (Å)	L_{cyl} (Å)	B	P	
**A200**	0.0218	150	15.5	0	0	0	0	0.000950	1.67	0.00137
**A300**	0.0133	191	25.4	0.00519	165	4.87	340	0.00972	1.40	0.00119
**P100**	0.0196	131	11.8	0	0	0	0	0.000774	1.72	0
**P150**	0.0170	178	19.3	0	0	0	0	0.000972	1.84	0

Supplementary Table 13 Summary of variable fitting parameters for SAXS data of unmixed particle populations.

	Sample	Spherical micelle models		Cylindrical micelle model				Power law		$background$
		φ_{s}Amine spheres	φ_{s}PEG spheres	φ_{cyl}	R_{cyl} (Å)	$\sigma_{R_{\mathrm{cyl}}}$ (Å)	L_{cyl} (Å)	B	P	
**Static**	**[A200•P100]**	0.000139	0.00143	0.00656	150	18.2	333	0.000727	1.65	0.00550
	**[A200•P150]**	0.0000115	0.00636	0.00437	172	37.5	469	0.00728	1.33	0.00421
	**[A300•P100]**	0.000979	0.00487	0.00356	186	24.1	669	0.0729	1.11	0.00421
	**[A300•P150]**	0.00514	0.000197	0.00414	194	12.2	1565	0	0	0.00421
**Time-resolved [A200•P100]**	2 s	-	-	0.00612	125	13.9	216	0.0446	1.05	0.00362
	4 s	-	-	0.00602	125	14.2	222	0.0883	0.946	0.00362
	6 s	-	-	0.00594	126	14.5	227	0.169	0.828	0.00362
	8 s	-	-	0.00590	126	14.7	231	0.593	0.616	0.00362
	10 s	-	-	0.00587	126	14.8	233	1.23	0.485	0.00362
	20 s	-	-	0.00572	127	15.3	247	7.87	0.168	0.00362
	30 s	-	-	0.00562	128	15.6	256	14.2	0.0613	0.00362
	40 s	-	-	0.00557	128	15.8	262	15.1	0.0736	0.00362
	50 s	-	-	0.00551	128	16.0	269	14.7	0.0638	0.00362
	100 s	-	-	0.00545	130	16.4	281	11.7	0.143	0.00362
	200 s	-	-	0.00546	132	17.2	290	10.6	0.159	0.00362
	300 s	-	-	0.00552	134	17.5	291	20.6	0.000000159	0.00362
	400 s	-	-	0.00566	136	17.7	283	27.1	0.000000159	0.00362
	500 s	-	-	0.00574	137	17.9	281	24.4	0.000000159	0.00362
	600 s	-	-	0.00577	138	18.1	282	23.1	0.000000159	0.00362

Supplementary Table 14 Summary of variable fitting parameters for SAXS data of mixed particle populations.

Sample	Mean sphere diameter, D_s (nm) $D_s = 2R_s$	Mean sphere aggregation number, N_s $N_s = \frac{\frac{4}{3}\pi R_s^3}{V_s}$	Mean cylinder cross-sectional diameter, D_{cyl} (nm) $D_{cyl} = 2R_{cyl}$	Mean cylinder length, L_{cyl} (nm)	Mean cylinder aggregation number, N_{cyl} $N_{cyl} = \frac{\pi R_{cyl}^2 L_{cyl}}{V_s}$	Mean cylinder aspect ratio $\frac{L_{cyl}}{D_{cyl}}$	Mean number of spheres fused to form a cylinder $\frac{N_{cyl}}{N_s}$	Volume fraction of cylinders present $\frac{\varphi_{cyl}}{\varphi_{cyl} + \varphi_s}$
**A200**	30.0	229	-	-	-	-	-	0
**A300**	38.3	317	32.9	34.0	313	1.03		0.281
**P100**	26.1	303	-	-	-	-	-	0
**P150**	35.7	513	-	-	-	-	-	0

Supplementary Table 15 Summary of values calculated for unmixed static samples using SAXS fitting parameters outlined in Supplementary Table 13.

Sample	Mean cylinder cross-sectional diameter, D_{cyl} (nm) $D_{cyl} = 2R_{cyl}$	Mean cylinder length, L_{cyl} (nm) $L_{cyl} = 2l_{cyl}$	Mean cylinder aggregation number, N_{cyl} $N_{cyl} = \frac{\pi R_{cyl}^2 L_{cyl}}{V_s}$	Mean cylinder aspect ratio $\frac{L_{cyl}}{D_{cyl}}$	Mean component starting particle aggregation number, N_{avg}	Mean number of particles fused to form a cylinder $\frac{N_{cyl}}{N_{avg}}$	Volume fraction of amine spheres present $\frac{\varphi_{s,amine}}{\Sigma\varphi}$	Volume fraction of PEG spheres present $\frac{\varphi_{s,PEG}}{\Sigma\varphi}$	Volume fraction of cylinders present $\frac{\varphi_{cyl}}{\Sigma\varphi}$
**[A200•P100]**	30.0	33.3	508	1.11	266	1.91	0.0171	0.176	0.807
**[A200•P150]**	34.4	46.9	808	1.36	371	2.18	0.00110	0.592	0.407
**[A300•P100]**	37.2	66.9	1180	1.80	311	3.80	0.104	0.518	0.378
**[A300•P150]**	38.8	156	2670	4.03	381	7.00	0.542	0.0207	0.437

Supplementary Table 16 Summary of values calculated for mixed static samples using SAXS fitting parameters outlined in Supplementary Table 14.

	Sample	Mean cylinder cross-sectional diameter, $\boldsymbol{D_{\text{cyl}}}$ (nm) $D_{\text{cyl}} = 2R_{\text{cyl}}$	Mean cylinder length, $\boldsymbol{L_{\text{cyl}}}$ (nm) $L_{\text{cyl}} = 2l_{\text{cyl}}$	Mean cylinder aggregation number, $\boldsymbol{N_{\text{cyl}}}$ $N_{\text{cyl}} = \frac{\pi R_{\text{cyl}}{}^2 L_{\text{cyl}}}{V_{\text{s}}}$	Mean cylinder aspect ratio $\frac{L_{\text{cyl}}}{D_{\text{cyl}}}$	Mean component starting particle aggregation number, N_{avg}	Mean number of particles fused to form a cylinder $\frac{N_{\text{cyl}}}{N_{\text{avg}}}$
Time-resolved [A200•P100]	2 s	25.0	21.6	230	0.865	266	0.864
	4 s	25.1	22.2	237	0.885	266	0.893
	6 s	25.2	22.7	244	0.904	266	0.918
	8 s	25.2	23.1	249	0.916	266	0.936
	10 s	25.3	23.3	252	0.922	266	0.949
	20 s	25.4	24.7	271	0.971	266	1.02
	30 s	25.5	25.6	283	1.00	266	1.06
	40 s	25.6	26.2	292	1.02	266	1.10
	50 s	25.7	26.9	302	1.05	266	1.13
	100 s	26.0	28.1	323	1.08	266	1.21
	200 s	26.5	29.0	345	1.10	266	1.30
	300 s	26.8	29.1	355	1.08	266	1.33
	400 s	27.1	28.3	353	1.04	266	1.33
	500 s	27.3	28.1	356	1.03	266	1.34
	600 s	27.5	28.2	362	1.02	266	1.36

Supplementary Table 17 Summary of values calculated for in-situ experiment using SAXS fitting parameters outlined in Supplementary Table 14.

5.4 Time-resolved analyses

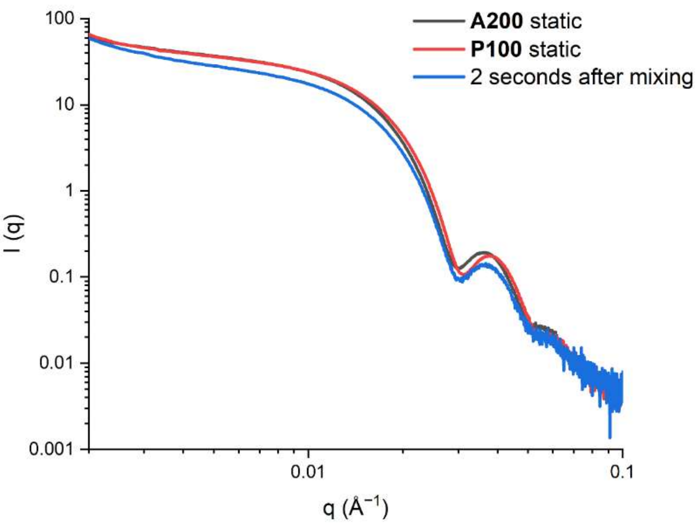

Supplementary Figure 34. SAXS plot for in-situ analysis two seconds after mixing. SAXS data for unmixed **A200** particles (black line), unmixed **P100** particles (red line) and after two seconds of mixing within the stopped-flow apparatus (blue line).

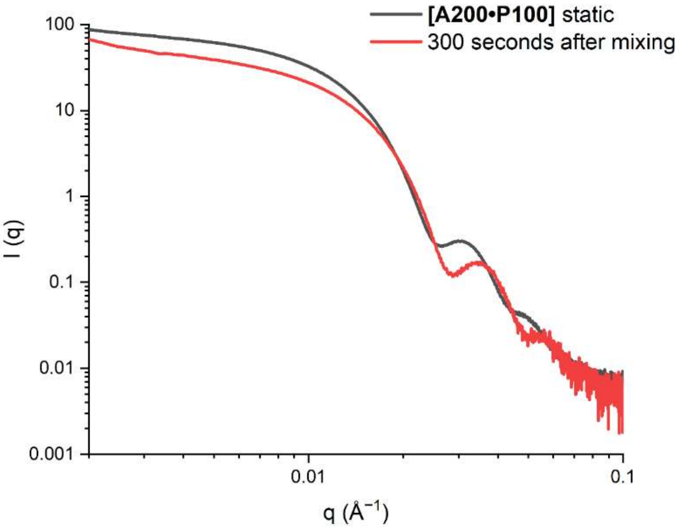

Supplementary Figure 35. SAXS plot for in-situ analysis 300 seconds after mixing. SAXS data for **[A200•P100]** particles formed by a standard laboratory experiment (black line) and after 300 seconds of mixing within the stopped-flow apparatus, when hetero-fusion was judged to be complete (red line).

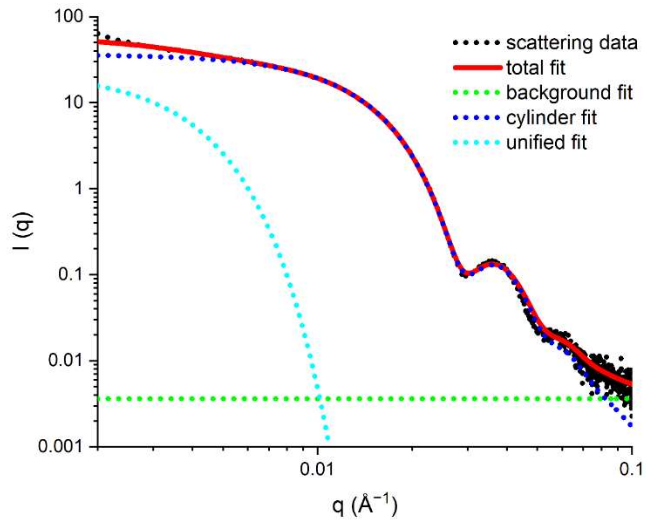

Supplementary Figure 36. SAXS plot depicting individual fits. SAXS data after 50 seconds of mixing within the stopped-flow apparatus (black scatter). Total fit (red line) obtained from a combination of a background fit (green scatter), cylindrical micelle fit (dark blue scatter) and a unified fit (light blue scatter).

6 Calculation of particles per unit volume

- **A200**, **A300**, **P100** and **P150** particles are synthesised by ROMPISA using a final concentration of 10 mg/mL of **NB-MEG**.

- Total moles of **NB-MEG** present in 1 mL = 0.045 mmol = 4.5×10^{-5} mol

- N_A = Avogadro's constant

A200 particles contain an average of 229 chains (N_{agg}) of poly(**NB-MEG**)$_{200}$

- Moles of **NB-MEG** per particle = number of **NB-MEG**/N_A = $(DP \times N_{agg})/N_A$ = 45,800/N_A = 7.6×10^{-20} mol

- Number of particles per mL = total moles of **NB-MEG**/moles of **NB-MEG** per particle = 5.9×10^{14}

P100 particles contain an average of 303 chains (N_{agg}) of poly(**NB-MEG**)$_{100}$

- Moles of **NB-MEG** per particle = number of **NB-MEG**/N_A = 30,300/N_A = 5.0×10^{-20} mol

- Number of particles per mL = total moles of **NB-MEG**/moles of **NB-MEG** per particle = 8.9×10^{14}

Therefore, as synthesised, there are ≈1.5× more **P100** particles per mL than **A200**

7 Increasing MEG DP: Characterisation data for hetero-fused samples [A200•P150], [A300•P100] and [A300•P150]

Characterisation Summary

Sample	$D_{h,DLS}$ /nm	PD_{DLS}	L_{TEM} /nm
**[A200•P150]**	85	0.10	59 ± 40
**[A300•P100]**	87	0.17	62 ± 47
**[A300•P150]**	242	0.26	120 ± 76

Supplementary Table 18 Characterisation of hetero-fused samples derived from **A300** and/or **P150**

DLS

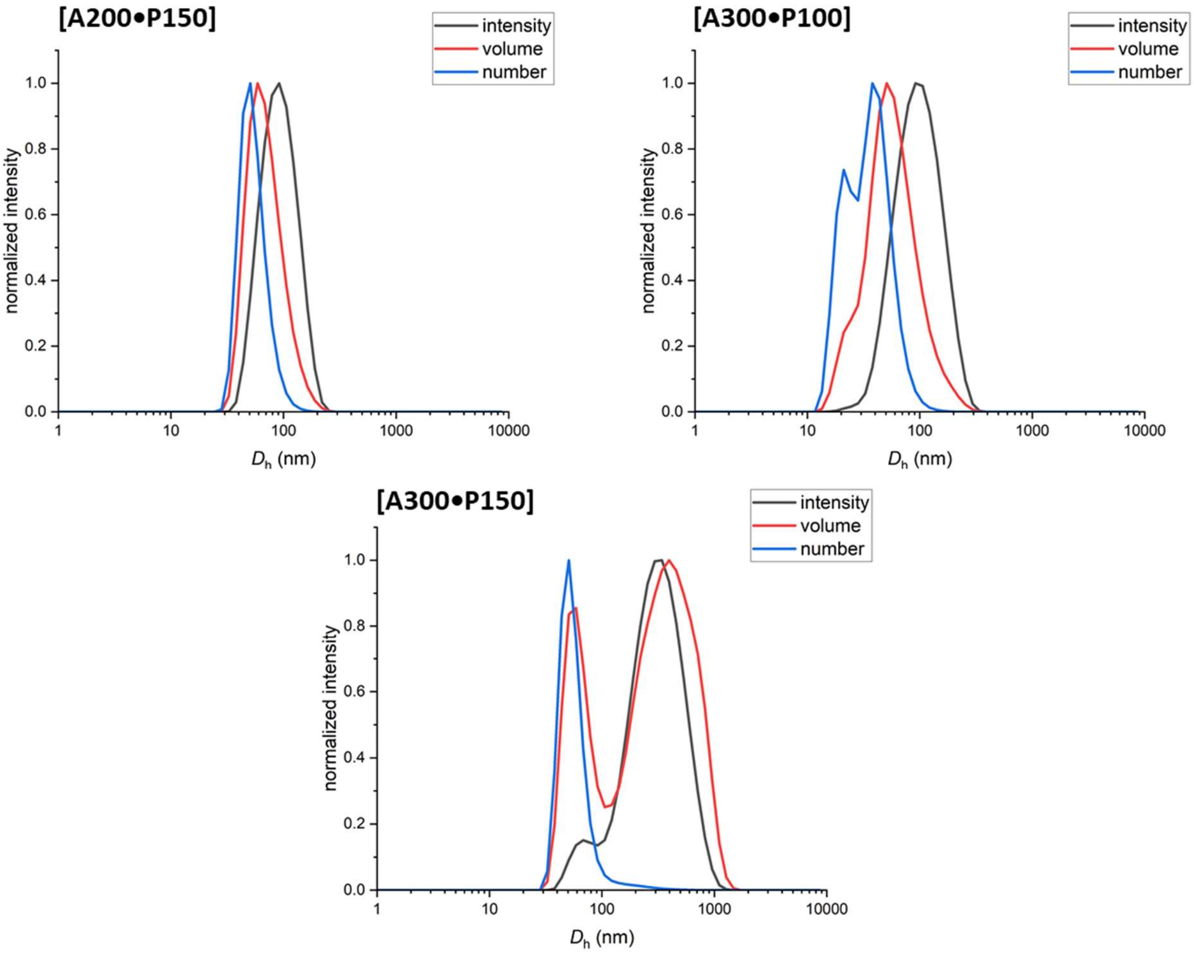

Supplementary Figure 37. DLS data for larger fused particles. DLS traces of **[A200•P150]**, **[A300•P100] and [A300•P150]** particles

Dry-state TEM

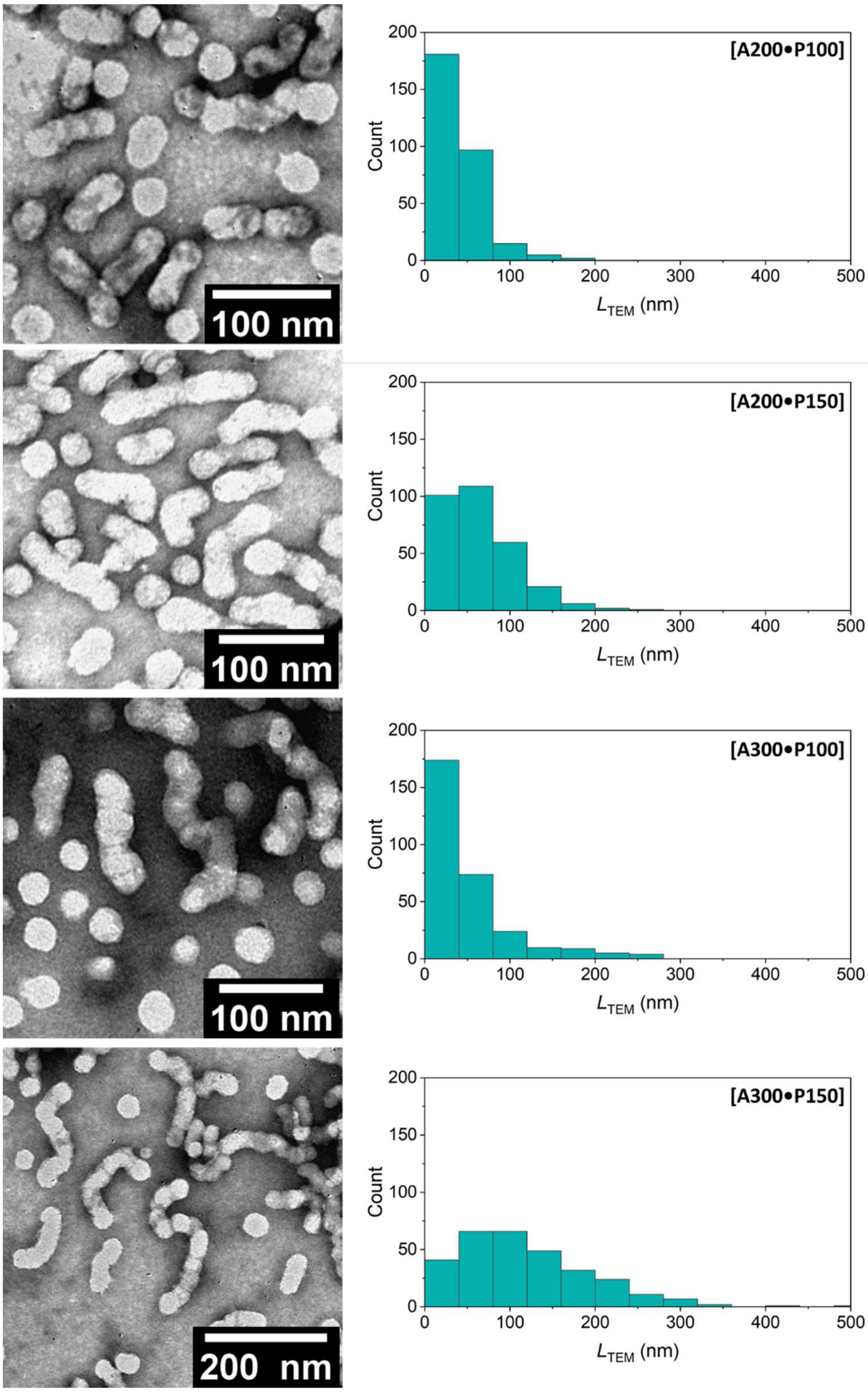

Supplementary Figure 38. TEM data for larger fused particles. Dry-state TEM images and histograms (300 particles per sample analysed) of **[A200•P100]** (histogram re-binned from Supplementary Figure 20 for comparison here)**, [A200•P150]**, **[A300•P100]** and **[A300•P150]**.

8 Synthesis of NB-FluoroMEG

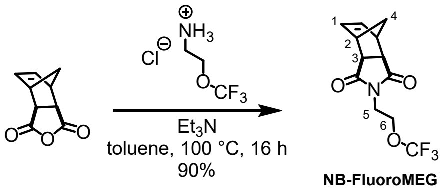

Supplementary Figure 39. Synthesis of NB-FluoroMEG. Synthetic scheme.

To a solution of cis-5-Norbornene-exo-2,3-dicarboxylic anhydride (330 mg, 2.0 mmol, 1.0 eq.) and 2-(trifluoromethoxy)ethylamine hydrochloride (363 mg, 2.2 mmol, 1.1 eq.) in toluene (5 mL) was added triethylamine (306 µL, 2.2 mmol, 1.1 eq.) dropwise. The resulting solution was stirred at 100 °C for 16 h. After cooling, EtOAc (20 mL) was added. This mixture was washed with 1 M aqueous HCl (20 mL × 2), brine (20 mL) and dried with $MgSO_4$. Removal of the volatiles under reduced pressure yielded **NB-FluoroMEG** (496 mg, 1.8 mmol, 90%) as a colorless oil.

^{1}H NMR (400 MHz, $CDCl_3$, 298 K): δ 6.50 – 6.09 (m, 2H, H_1), 4.14 (t, J = 5.6 Hz, 2H, H_6), 3.80 (t, J = 5.6 Hz, 2H, H_5), 3.29 (t, J = 1.8 Hz, 2H, H_2), 2.72 (d, J = 1.4 Hz, 2H, H_3), 1.52 (dt, J = 10.0, 1.6 Hz, 1H, H_4), 1.35 – 1.21 (m, 1H, H_4).

^{19}F NMR (377 MHz, $CDCl_3$, 298 K): δ −61.1 (s)

^{13}C NMR (101 MHz, $CDCl_3$, 298 K): δ 177.75, 137.95, 121.53 (q, J = 253.9 Hz), 63.21 (q, J = 3.3 Hz), 47.98, 45.40, 42.73, 37.36.

HRMS (CI^+) Calculated for $C_{12}H_{13}F_3NO_3$ $[M+H]^+$ 276.0842, found 276.0841

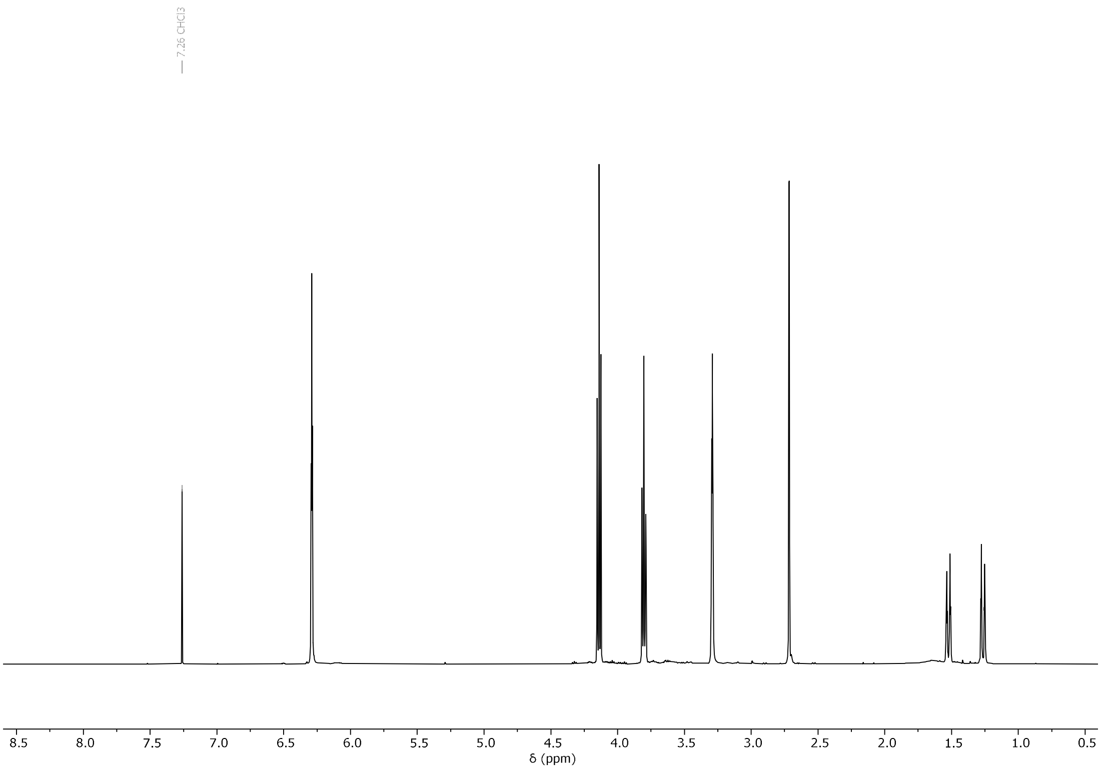

Supplementary Figure 40. ^{1}H NMR spectrum of NB-FluoroMEG. ^{1}H NMR (400 MHz, $CDCl_3$, 298 K) of **NB-FluoroMEG**

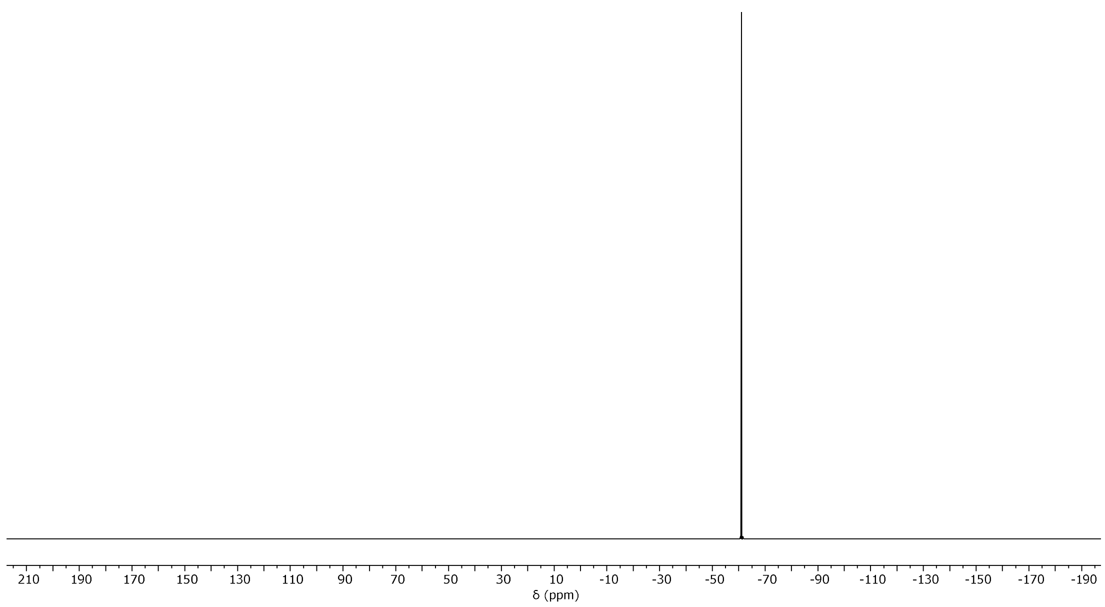

Supplementary Figure 41. ^{19}F NMR spectrum of NB-FluoroMEG. ^{19}F NMR (377 MHz, $CDCl_3$, 298 K) of **NB-FluoroMEG**

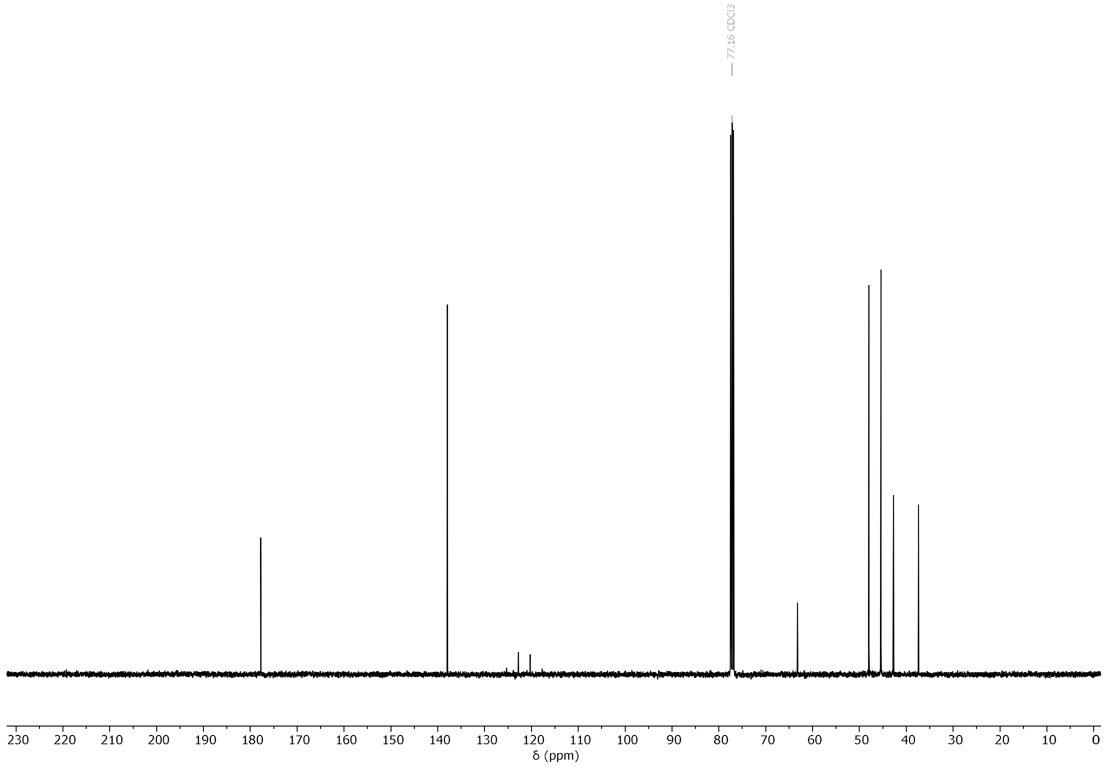

Supplementary Figure 42. ^{13}C NMR spectrum of NB-FluoroMEG. ^{13}C NMR (101 MHz, $CDCl_3$, 298 K) of **NB-FluoroMEG**

9 Synthesis, characterisation and hetero-fusion of P(NB-amine)$_{11}$-*block*-P(NB-FluoroMEG)$_{200/300}$ (AF200)

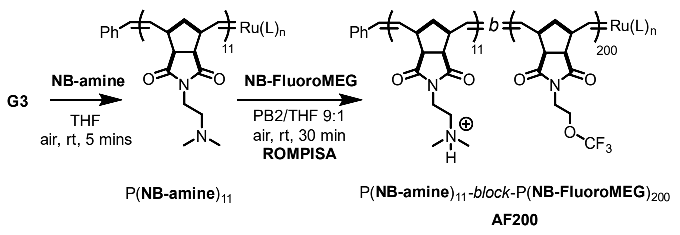

Supplementary Figure 43. Synthesis of AF200. ROMPISA synthetic scheme.

A solution of **NB-amine** (11.6 mg, 50 µmol, 11 eq.) in 900 µL filtered THF was rapidly added to a solution of **G3** (3.3 mg, 4.5 µmol, 1.0 eq.) in 100 µL filtered THF, contained within a 2 mL glass vial equipped with a stirrer bar. The resulting solution was stirred rapidly for five minutes.

An aliquot (50 µL) of a macroinitiator solution in THF was dispensed into a 2 mL glass vial containing a stirrer bar. A solution of **NB-FluoroMEG** (12.4 mg, 45 µmol, 200 eq.) in 900 µL of PB2 and 50 µL filtered THF was added rapidly to the vial (final solids concentration = 1.24 wt%). The resulting mixture was thoroughly mixed by drawing up the entire volume into the pipette tip and ejecting the liquid back into the vial three times. The ROMPISA polymerisation were stirred at 300 rpm for 30 minutes to give the **AF200** nanoparticle population. This was analysed by SEC, DLS and TEM.

Characterisation summary

Sample	$D_{h,DLS}$ /nm	PD_{DLS}	L_{TEM} /nm
**AF200**	73[a]	0.30[a]	25 ± 3[b]
**[AF200•P100]**	364[a]	0.46[a]	38 ± 12[b]

[a]A small number (<1% of total) of anomalously large particles (observed by TEM) obscure the $D_{h,DLS}$ and associated PD_{DLS} values. [b]Anomalously large particles omitted when calculating L_{TEM}.

Supplementary Table 19 Characterisation of AF200 polymers and particles.

DLS

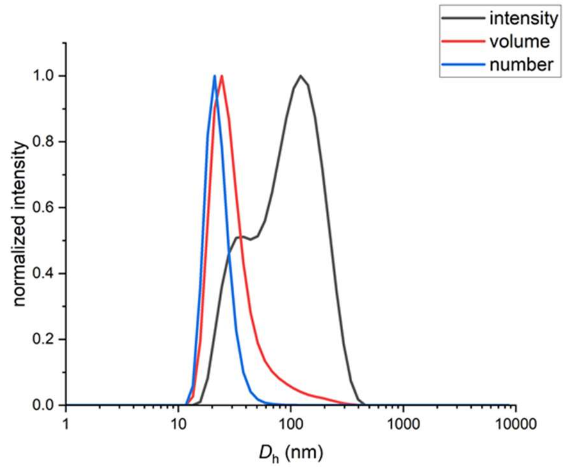

Supplementary Figure 44. DLS data for AF200. DLS traces of **AF200** particles

TEM

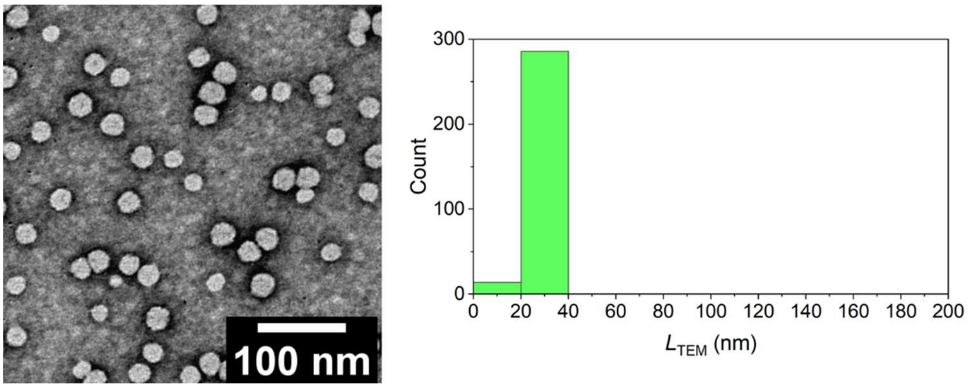

Supplementary Figure 45. TEM data for AF200. Dry-state TEM image and histogram (300 particles analysed) of **AF200** particles

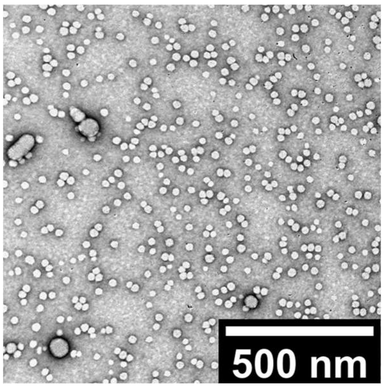

Supplementary Figure 46. TEM data for AF200 depicting large particles. Dry-state TEM image of **AF200** particles showing anomalously large particles

Hetero-fusion

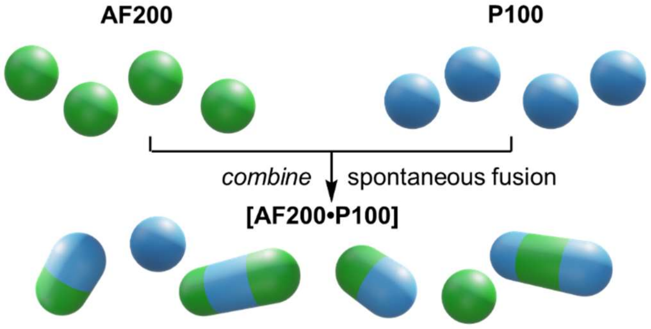

Supplementary Figure 47. Hetero-fusion between AF200 and P100. Cartoon depicting mixing to produce fused particles.

An aliquot of **AF200** was added to a 2 mL glass vial by pipette. A second aliquot of **P100** was added into the vial to produce a final volume of 200 µL. The resulting mixture was thoroughly mixed by drawing up the entire volume into the pipette tip and ejecting the liquid back into the vial three times. The mixture was left for 30 minutes without stirring before analysis.

DLS

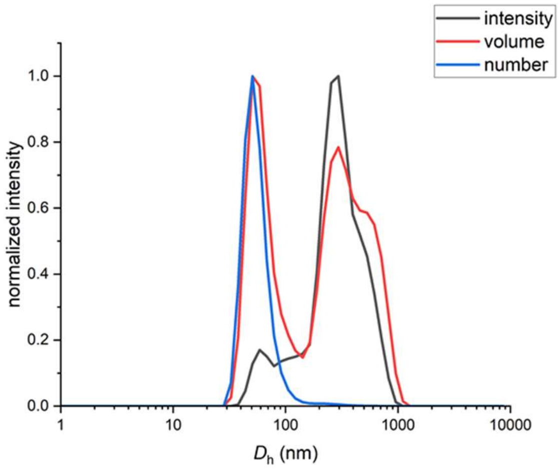

Supplementary Figure 48. DLS data for [AF200•P100]. DLS traces of **[AF200•P100]** particles

TEM

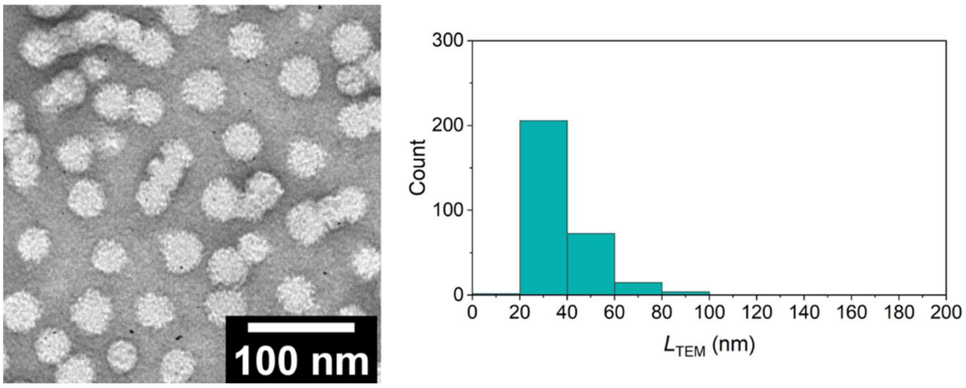

Supplementary Figure 49. TEM data for [AF200•P100]. Dry-state TEM image and histogram (300 particles analysed) of **[AF200•P100]** particles

10 Cryo-STEM-EELS analysis of AF200 and [AF200•P100] particles

10.1 Experiment description

A sample of **AF200** or **[AF200•P100]** was diluted 100× with PB2. Next, 5 µL of diluted sample was deposited onto a lacey carbon grid followed by blotting for approximately five seconds. The grid was then plunged into a pool of liquid ethane, cooled using liquid nitrogen, to vitrify the sample using a ThermoFisher Vitrobot. Transfer into a pre-cooled cryo-TEM holder (Gatan 626 Cryo Holder) was performed under liquid nitrogen temperatures prior to microscopic analysis. The sample was transferred into a Thermo Fisher Scientific Spectra 300 microscope (University of Cambridge) equipped with a high brightness 'X-FEG' electron source and operated at 300 keV electron beam energy. The beam convergence semi-angle was set to 0.5 mrad. STEM imaging was carried out using the Panther detection system for simultaneous angle-resolved dark field and bright field image acquisition. Electron energy loss spectroscopy (EELS) data was acquired using a Gatan Continuum HR spectrometer, operated with a dispersion of 0.3 eV/channel. Regions suitable for analysis were identified using a combination of bright-field and annular dark-field imaging. This analysis also confirmed that morphological changes are not introduced during electron beam exposure. Cryo-STEM-EELS spectrum images (i.e. EEL spectra acquired at every probe position in a scanned field of view) were acquired for the selected regions with a spatial resolution (pixel size) of 7 nm. Sub-pixel scanning of the pixel area during EELS acquisition was implemented to spread the fluence across the entire pixel area. The parameters provided sufficient spatial resolution to resolve the nanoparticles while minimising electron beam-induced damage.

Technique	Pixel size	Probe current	Dwell time	Cumulative electron fluence per scan
ADF	1.4 nm	5 pA	10 µs	1.5 e^{-} $Å^{-2}$
EELS	7 nm	5 pA	0.1 s	640 e^{-} $Å^{-2}$

Supplementary Table 20 Parameters for STEM experiments

10.2 Data analysis

To further control analyses, EELS measurements to evaluate fusion in **[AF200•P100]** particles were carried out over >10 fused regions across multiple separate areas where particles were observed within ice sufficiently thin for electron transparency (Figures S47-S48). The datasets were first aligned to remove any shifts in the spectrum on the camera by using cross-correlation based methods. This aligned the zero-loss peak throughout the spectrum image dataset to subpixel precision. Then, intensity artefacts arising from X-rays striking the detector camera were removed. This was achieved using routines in the HyperSpy 1.7.3 (https://zenodo.org/records/7263263) software package with interpolation with Poissonian noise after spike removal. Then spectra were rebinned by a factor of 4 along the energy axis to reduce noise in the spectra.

Next, an independent component analysis (ICA) blind source separation algorithm was applied to the core-loss STEM-EELS data. ICA decomposes the hyperspectral dataset (or 'data-cube') into a small number of components each associated with a map. The intensities in these maps give the coefficients for reconstructing the dataset as a linear combination of the components. The ICA decomposition

consistently retrieved three components in these datasets: (1) One component showed signals at the C *K* edge, N *K* edge, and F *K* edge; (2)-(3) the other two components showed (2) O *K* edge intensity and (3) varying background. The C, N, and F containing component corresponded to localised intensity within the nanoparticles. The map corresponding to this component was selected as an overview map of the polymer contribution. Similar approaches have been successfully applied to separate chemical phases and background features in core-loss EELS.[51–53] Here, we use this map only as a map of the polymer distribution rather than seeking to directly interpret the spectra factors recovered by ICA. This overview map allowed all nanoparticles, labelled or unlabelled, to be located. Nanoparticle location information then provided the basis for identifying regions of interest (ROIs) for determining fluorine distribution. Selected area spectra were extracted with power law background fitting applied across a 'pre-edge' energy window at approximately 610 to 680 eV energy loss. The EELS data were then plotted for selected ROIs. The ROIs were set to 21×21 nm or 3×3 pixels in for datasets shown in Supplementary Figures 45-47 or 28×28 nm or 4×4 pixels for the field of view shown in Supplementary Figure 48. For the **AF200** (unfused) sample, all particles contain fluorine. ROIs around the edge of a ‘cluster’ of particles were selected (Fig. S45), as they produced a stronger fluorine signal. We attribute the higher signal in these regions to larger projected size of nanoparticles. This is possibly due to the vitrification process in these regions Across regions with varying amorphous ice thickness, there will be an optimal thickness for maximum signal-to-noise ratio in EELS. EEL spectra were compiled for 14 ROIs (Fig. S45). Signal above the background level and past the fluorine *K* edge was observed for 9 particles (64%).

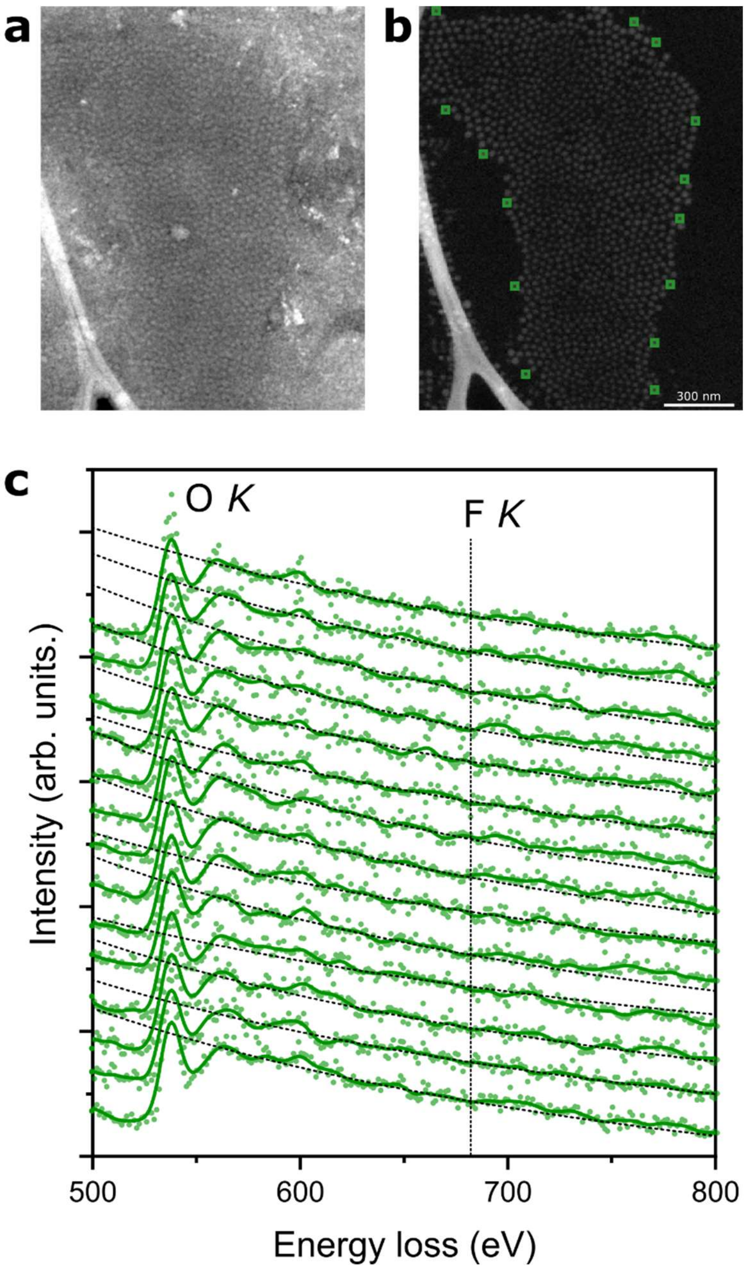

Supplementary Figure 50. EELS analysis of AF200 particles. **a** ADF image, **b** ICA carbon map with selected ROIs indicated by green boxes. **c** EELS spectra of ROIs.

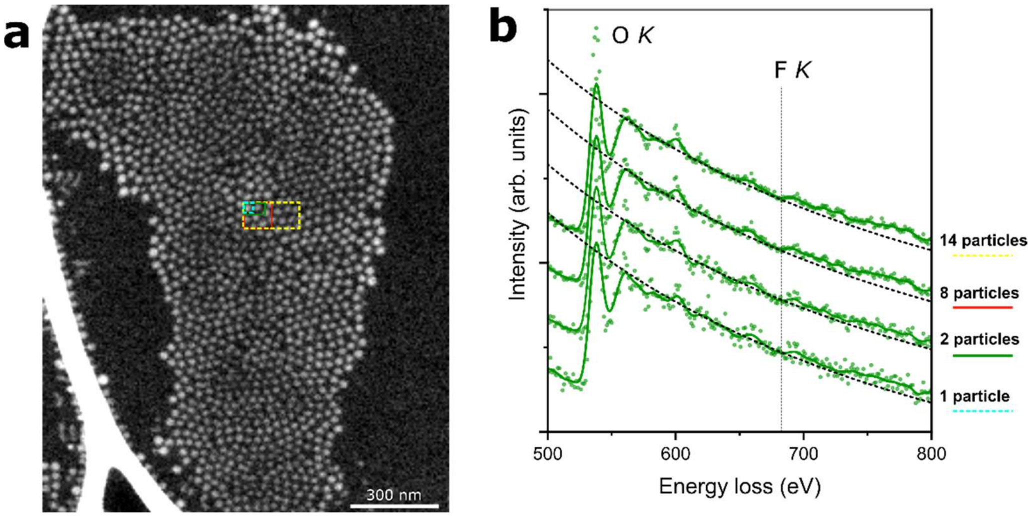

Supplementary Figure 51. Evaluation of signal-to-noise ratio and detection limits for AF200 particles. a, ICA carbon map with regions of interest comprising 1, 2, 8, and 14 particles overlaid. **b**, Area-averaged spectra from the four regions of interest, illustrating the improved signal-to-noise ratio with increased averaging. The F *K* edge onset feature is visible, particularly with gaussian smoothing applied (solid line), but is close to the noise level of the tail of the O *K* edge at 1 particle. Summing 8-14 particles removes uncertainty from the noise and shows a clear step onset at the F *K* edge. This analysis confirms that the summation of 15 particles assigned as unlabelled in the **[AF200•P100]** sample would be expected to show a defined onset at the F *K* edge (matched experimental conditions) if these were F-labelled particles. No such onset was observed in Fig. S47 for unlabelled regions, providing strong evidence of robust detection of substantially uneven F distribution in **[AF200•P100].**

For the **[AF200•P100]** sample, unfused **AF200**, unfused **P100** and hetero-fused particles are present. ROIs were selected from adjacent positions in fused particles across two particle clusters (area 1: Figs. S47 and 2: S48); all ROIs had a least one 'neighbour' ROI. This ensured that, if the fused particles adopt a patterned structure, both labelled and unlabelled regions were selected. Overall, 28 ROIs were analysed from two areas of the TEM grid and 13 were judged to contain a fluorine signal (46%). To ensure the observed fluorine signal was not an artefact, the spectra for the fluorine-containing and fluorine absent ROIs were summed. The resultant spectra (Figs. S47c,d, S48c) clearly demonstrate the presence of fluorine in alternate positions along fused particles. This indicates (1) fused particles are formed from both **AF200** + **P100** particles and (2) fluorine is not evenly distributed within fused particles. These findings do not specify whether the interfaces between fused **AF200** and **P100** particles are abrupt or smooth, but provide clear evidence for varying distribution of the F-labelled contribution.

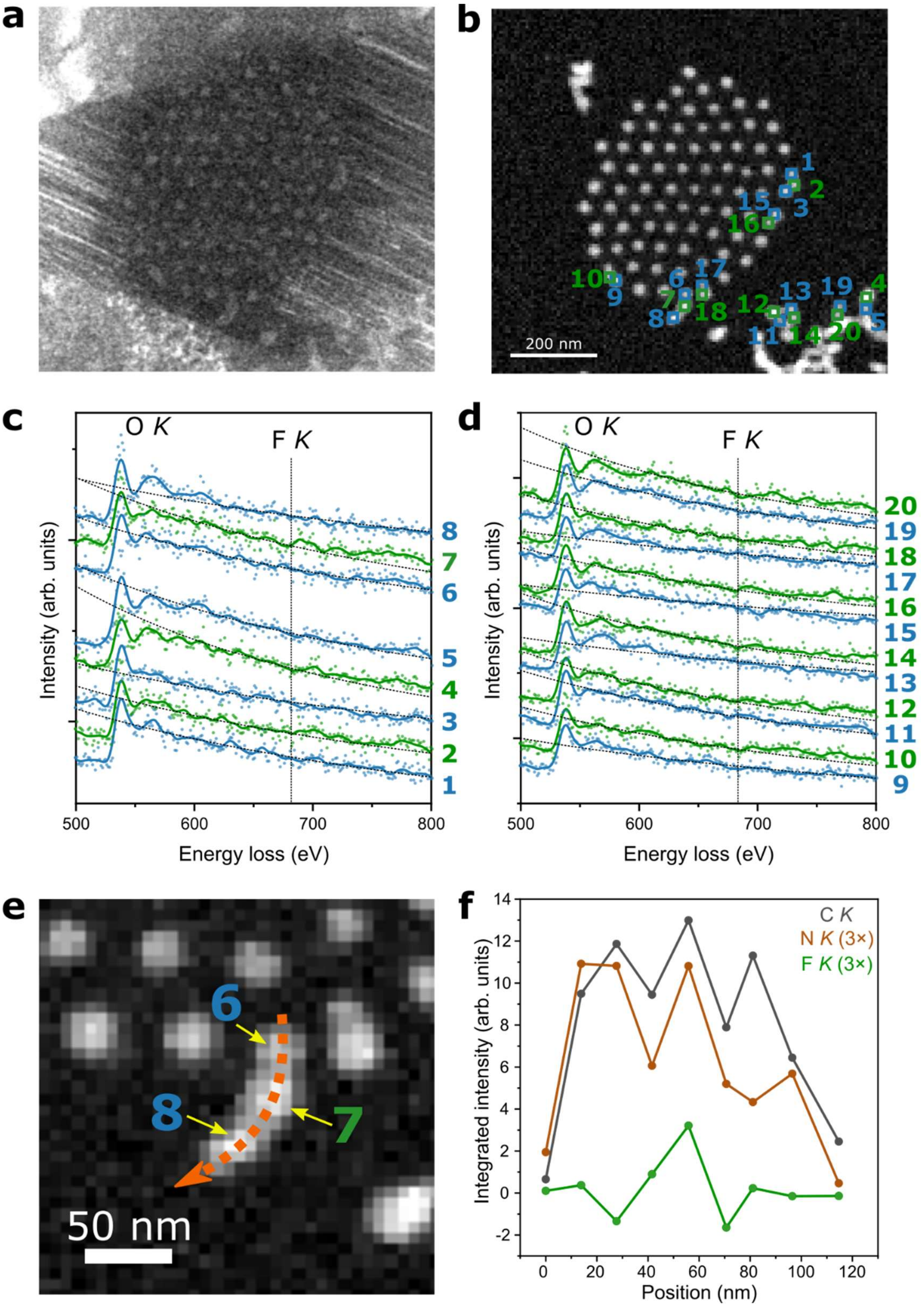

Supplementary Figure 52. Analysis of [AF200•P100] particles, area 1. a ADF image, **b** ICA carbon map. Fluorine containing ROIs are indicated by green boxes and fluorine absent ROIs are indicated by blue boxes. **c and d** EELS spectra of ROIs. Green = fluorine signal observed, blue = fluorine signal absent. **e** Highlighted subregion from **b** used for extraction of a line profile. The orange dashed arrow marks the selected line and the yellow arrows mark the positions of the corresponding spectra 6, 7, and 8 shown in **c. f** Line profiles showing the integrated intensities at the C *K*, N *K*, and F *K* edges from 14x14 nm (2x2 pixel) positions along the line. Power law background fitting was

applied to remove the background and the edges were integrated across 50 eV energy loss windows. The N *K* and F *K* data are shown multiplied by a factor of three for ease of comparison with the C *K* position-dependent intensities. The F *K* signal is near the limit of detection, resulting in baseline oscillations due to imperfect background fitting and limited counts in the integrated edge. Together with spectra in **c**, the line profile illustrates the detection of F at position 7 and no detection F at positions 6 or 8.

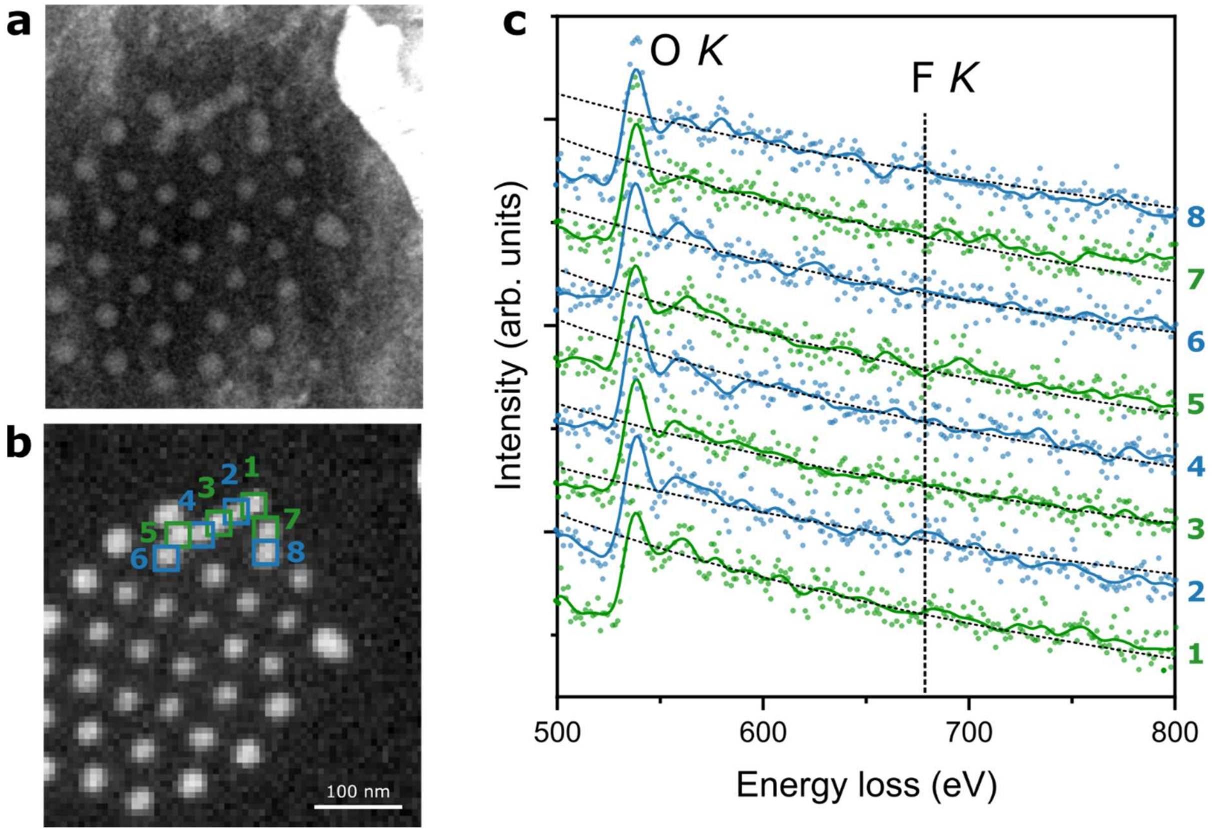

Supplementary Figure 53 Analysis of [AF200•P100] particles, area 2. a ADF image, **b** ICA carbon map. Fluorine containing ROIs are indicated by green boxes and fluorine absent ROIs are indicated by blue boxes. **c and d** EELS spectra of ROIs. Green = fluorine signal observed, blue = fluorine signal absent.

11 Additional control experiments

11.1 Storage stability study

After two weeks, only spherical particles were observed by TEM for a **[A100•P50]** sample (Fig. S49). However, the average particle diameter (L_{TEM} = 35 ± 6 nm) was larger than for the starting **A200** and **P100** particles, indicating that fused particles likely collapse into a spherical shape over time. The hetero-fused structures are therefore kinetically trapped when first formed.

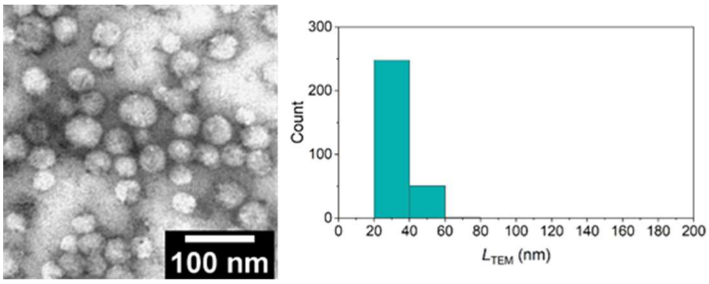

Supplementary Figure 54. Assessing aging of fused particles. [A200•P100] particles aged for two weeks. Particles stained with AGR1000 UA-Zero stain

11.2 Attempted hetero-fusion of A200 and P100 at low temperature.

Being a kinetically controlled process, hetero-fusion of **A200** and **P100** should be inhibited at low temperature. Therefore, to ascertain the effects of lowering the temperature, we combined pre-cooled **A200** and **P100** particles in a 1:1 volume ratio at 0 °C in an ice bath. As evident from the SAXS data (section 5.4), the hetero-fusion process is complete within five minutes at room temperature (rt). Fusion at 0 °C was quenched after five minutes by adding buffer. This halts the fusion process by diluting THF, which inhibits chain mobility. TEM analysis (Fig. S50) shows that fusion is almost completely suppressed by lowering the temperature (L_{TEM} = 31 ± 5 nm @ 0 °C; 45 ± 23 nm @rt).

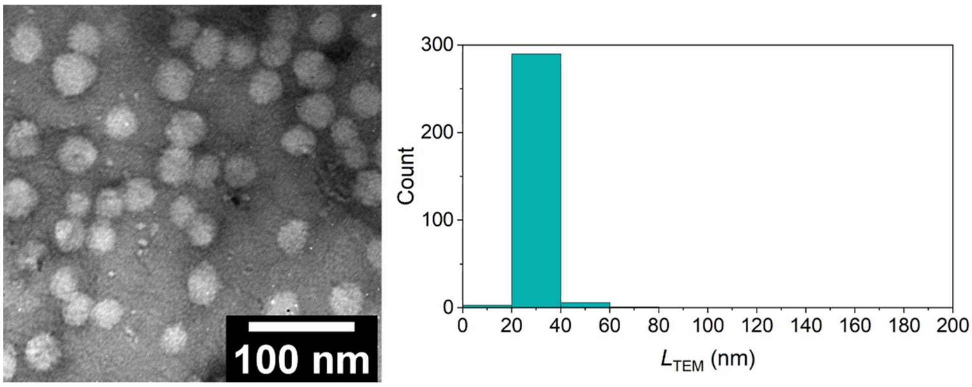

Supplementary Figure 55. Low temperature experiment. Attempted hetero-fusion of **A200** and **P100** particles at 0 °C. Particles stained with AGR1000 UA-Zero stain.

11.3 Increasing the THF content during hetero-fusion

THF acts as a plasticiser in the fusion process. It can be regarded as a 'catalyst' for chain mobility. It also needs to be present to dissolve **G3** and hence form the corona-forming polymer prior to the addition of **NB-MEG** in buffer. Therefore, whilst THF cannot be removed from the process, more can be added.

To explore the effect of adding more THF, the standard fusion experiment between **A200** and **P100** particles in a 1:1 volume ratio in the presence of 50% THF. This was achieved by adding additional THF immediately after mixing the particles. This results (Fig. S51) in a greater extent of fusion (L_{TEM} = 78 ± 85 nm @ 50% THF [note positive skew]; 45 ± 23 nm @10% THF). Evidently, more THF lowers the activation energy for fusion for a greater number of particles. At higher proportions of THF the polymer becomes soluble.

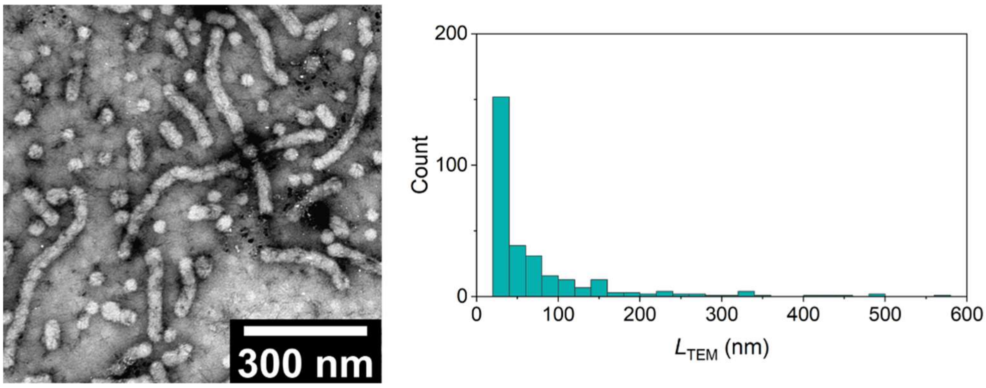

Supplementary Figure 56. Fusion with greater THF content. Hetero-fusion of **A200** and **P100** particles at with 50% THF content. Particles stained with AGR1000 UA-Zero stain.

11.4 Combining non-fusogenic particle populations A100 and P50

A100 and **P50** particles do not fuse when mixed together (Fig. S52). This is because in both populations the DP of the P(**NB-MEG**) block is too low to provide sufficient driving force for either homo- or hetero-fusion.

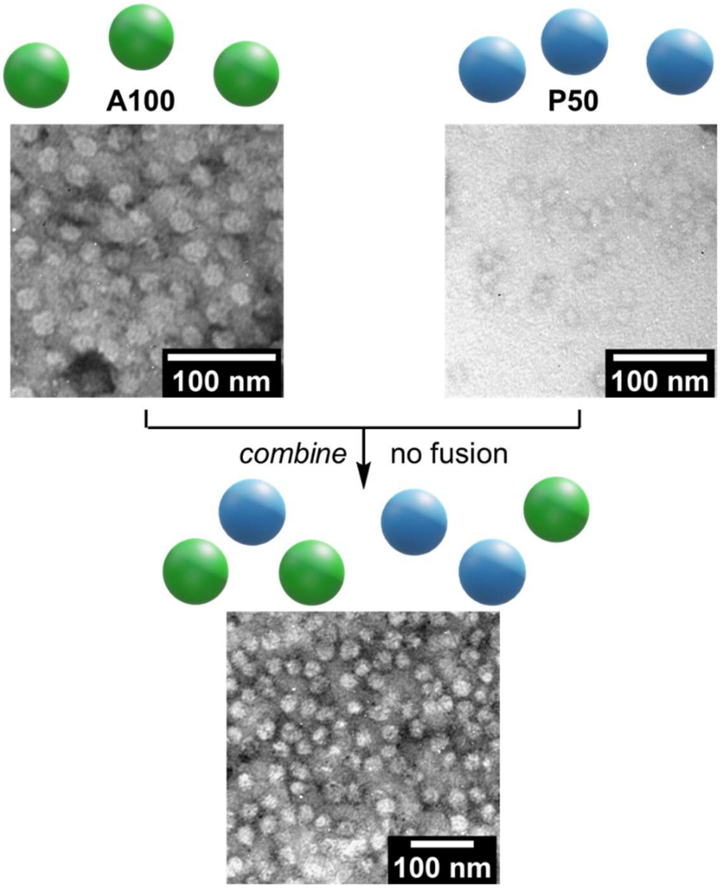

Supplementary Figure 57. Mixing of particles with low NB-MEG DP. Attempted hetero-fusion of **A100** and **P50** particles. Particles stained with AGR1000 UA-Zero stain. Note **P50** particles do not stain well due to their small size

11.5 Preliminary study of hetero-fusion at neutral pH

Particles containing **NB-COOH** within the corona were used to explore whether negatively charged particles could also participate in hetero-fusion at neutral pH (Fig. S53). In order to form a colloidally stable system, **NB-COOH** needed to be combined with **NB-PEG** to reproducibly form colloidally stable particles. Therefore, negatively charged particles, **C200**, were made of the polymer P(**NB-PEG**)$_{11}$-*block*-P(**NB-COOH**)$_{5}$-*block*-P(**NB-MEG**)$_{200}$ at pH 7. These particles were spherical prior to hetero-fusion.

Given **NB-PEG** was already situated in the negatively charged particles, it was most appropriate to study hetero-fusion to a positively charged particle population, rather than neutral (i.e., not **P100**).

Therefore, as **A200** particles were colloidally unstable above pH 4, an additional population of positively charged particles, **D200**, that were stable at pH 7 were made. These contained P(**NB-PEG**)$_{11}$-*block*-P(**NB-Amine**)$_{5}$-*block*-P(**NB-MEG**)$_{200}$. These particles were also spherical prior to hetero-fusion.

Combining **C200** and **D200** resulted in **[C200•D200]**, clearly showing fusion also occurs in this system.

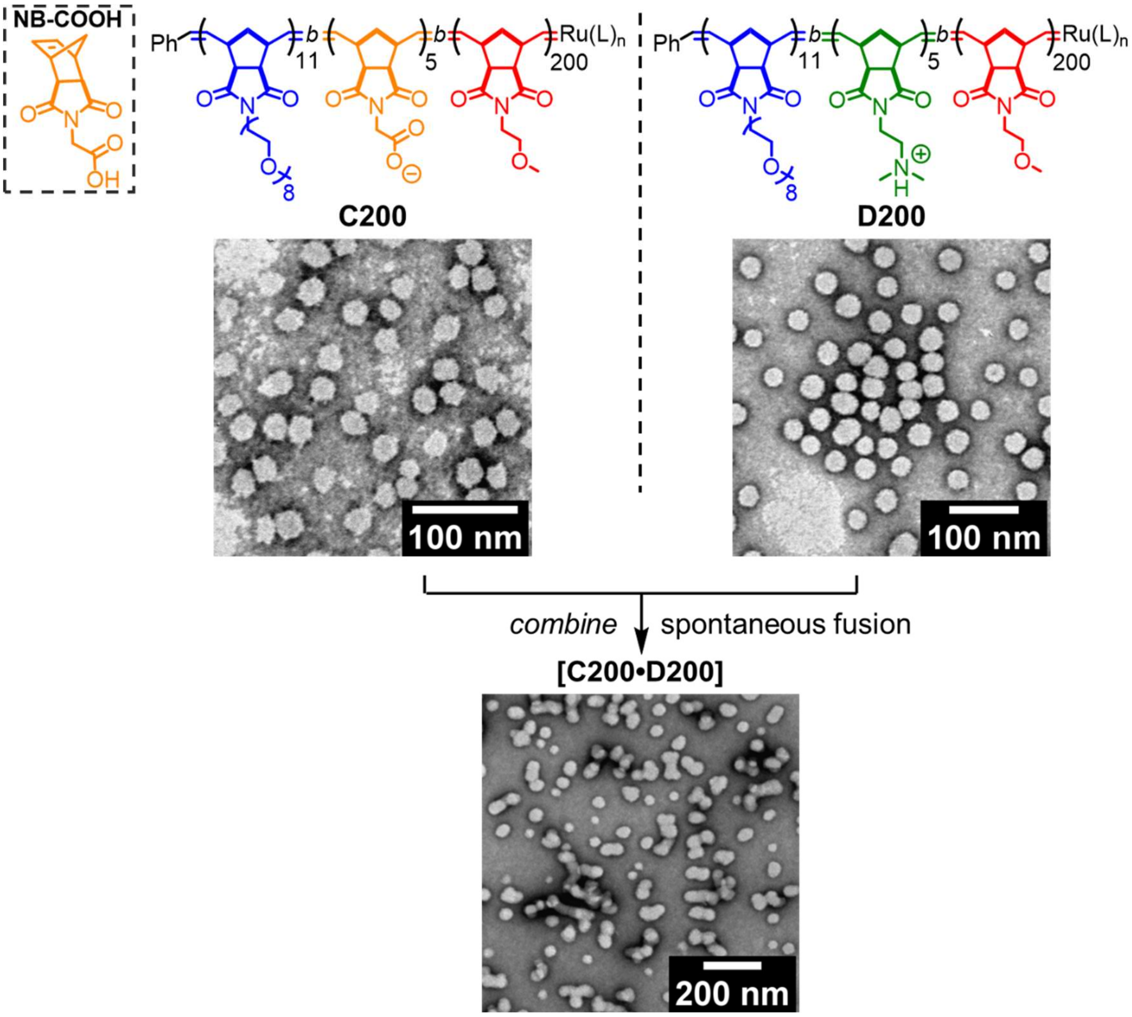

Supplementary Figure 58. Fusion involving negatively charged particles. Hetero-fusion between **C200** and **D200** at neutral pH.

12 Supplementary References

1. Radzinski, S. C. *et al.* Tapered Bottlebrush Polymers: Cone-Shaped Nanostructures by Sequential Addition of Macromonomers. *ACS Macro Lett.* **6**, 1175–1179 (2017).
2. Varlas, S. *et al.* Predicting Monomers for Use in Aqueous Ring-Opening Metathesis Polymerization-Induced Self-Assembly. *ACS Macro Lett.* **8**, 466–472 (2019).
3. Fielden, S. D. P., Derry, M. J., Miller, A. J., Topham, P. D. & O'Reilly, R. K. Triggered Polymersome Fusion. *J. Am. Chem. Soc.* **145**, 5824–5833 (2023).
4. Filik, J. *et al.* Processing two-dimensional X-ray diffraction and small-angle scattering data in DAWN 2. *J. Appl. Crystallogr.* **50**, 959–966 (2017).
5. Odian, G. *Principles of Polymerization*, 4th edn Page 76 (John Wiley & Sons, Inc 2004).
6. Ilavsky, J. & Jemian, P. R. Irena: tool suite for modeling and analysis of small-angle scattering. *J. Appl. Crystallogr.* **42**, 347–353 (2009).
7. Pedersen, J. S. Form factors of block copolymer micelles with spherical, ellipsoidal and cylindrical cores. *J. Appl. Crystallogr.* **33**, 637–640 (2000).
8. Sponchioni, M. *et al.* Probing the mechanism for hydrogel-based stasis induction in human pluripotent stem cells: is the chemical functionality of the hydrogel important? *Chem. Sci.* **11**, 232–240 (2020).
9. Pedersen, J. S. & Gerstenberg, M. C. The structure of P85 Pluronic block copolymer micelles determined by small-angle neutron scattering. *Colloids Surf. A Physicochem. Eng. Asp.* **213**, 175–187 (2003).
10. Pedersen, J. S., Svaneborg, C., Almdal, K., Hamley, I. W. & Young, R. N. A Small-Angle Neutron and X-ray Contrast Variation Scattering Study of the Structure of Block Copolymer Micelles: Corona Shape and Excluded Volume Interactions. *Macromolecules* **36**, 416–433 (2003).
11. Pedersen, J. S. & Schurtenberger, P. Scattering Functions of Semiflexible Polymers with and without Excluded Volume Effects. *Macromolecules* **29**, 7602–7612 (1996).
12. Warren, N. J., Mykhaylyk, O. O., Mahmood, D., Ryan, A. J. & Armes, S. P. RAFT aqueous dispersion polymerization yields poly(ethylene glycol)-based diblock copolymer nano-objects with predictable single phase morphologies. *J. Am. Chem. Soc.* **136**, 1023–1033 (2014).
13. de la Pena, F. *et al.* Mapping titanium and tin oxide phases using EELS: an application of independent component analysis. *Ultramicroscopy* **111**, 169–176 (2011).
14. Collins, S. M., Fernandez-Garcia, S., Calvino, J. J. & Midgley, P. A. Sub-nanometer surface chemistry and orbital hybridization in lanthanum-doped ceria nano-catalysts revealed by 3D electron microscopy. *Sci. Rep.* **7**, 5406 (2017).
15. Collins, S. M. *et al.* Subwavelength Spatially Resolved Coordination Chemistry of Metal-Organic Framework Glass Blends. *J. Am. Chem. Soc.* **140**, 17862–17866 (2018).
